# Hic-5 promotes the progression of nonalcoholic steatohepatitis by regulating hepatocellular fatty acid metabolism through the PTEN/PGE2/EP4 axis

**DOI:** 10.1186/s43556-026-00409-4

**Published:** 2026-02-09

**Authors:** Zhiwei Huang, Peng Tan, Boyuan Gu, Shenglu Liu, Han Li, Jiatong Chen, Bingyu Ren, Lei Sun, Jian Wen, Yu Li, Wenguang Fu

**Affiliations:** 1 Department of General Surgery (Hepatobiliary Surgery), Department of Biliary-Pancreatic Center, The Affiliated Hospital, Southwest Medical University, Sichuan Province, 25 Taiping Street, Luzhou, 646000 China; 2 Metabolic Hepatobiliary and Pancreatic Diseases Key Laboratory of Luzhou City, Academician (Expert) Workstation of Sichuan Province, The Affiliated Hospital, Southwest Medical University, Luzhou, 646000 China; 3 CAS Key Laboratory of Nutrition, Metabolism and Food Safety, Shanghai Institute of Nutrition and Health, University of Chinese Academy of Sciences, Chinese Academy of Sciences, Xuhui District, No. 320 Yueyang Road, Shanghai, 200031 China

**Keywords:** Nonalcoholic steatohepatitis, Hic-5, Hepatic stellate cells, Hepatocytes, Fatty acid metabolism

## Abstract

Nonalcoholic steatohepatitis (NASH) is a metabolic disease characterized by hepatic steatosis and inflammation among other features. Dysregulated lipid metabolism is crucial in the pathogenesis of NASH. However, its regulatory mechanisms remain intricate and poorly elucidated. Hepatic stellate cells (HSCs) have been reported to contribute to hepatocellular lipid metabolism dysregulation and aggravate NASH progression. However, the potential mechanisms remain unclear. Here, we demonstrate that hydrogen peroxide-inducible clone 5 (Hic-5), which is highly expressed in HSCs within the liver, is elevated in NASH patients and mouse models. Hic-5 deficiency alleviates hepatic steatosis, and liver metabolomics revealed reduced fatty acid levels. Meanwhile, RNA-sequencing revealed that Hic-5 deficiency increases AMPK phosphorylation. Additionally, HSC-specific overexpression of Hic-5 exacerbates NASH severity. Co-culture experiments indicated that Hic-5 increases hepatocellular fatty acid synthesis. Cellular transcriptomic analysis and validation revealed that prostaglandin E2 (PGE2), secreted by HSCs, mediates hepatocellular fatty acid synthesis. Mechanistically, the N-terminal domain of Hic-5 binds c-Src, leading to phosphorylation of PTEN, which is bound to the C-terminal domain. This event subsequently induces phosphorylation and nuclear translocation of the transcription factor SP1, ultimately increasing PGE2 secretion. Finally, Hic-5 promotes hepatocellular fatty acid synthesis by activating the PGE2-EP4 axis. Pharmacological inhibition of EP4 in HSC-specific Hic-5 overexpression mice fed with HFD diet (HFD) significantly attenuated NASH progression. These findings increase our understanding of molecular mechanisms linking hepatic lipid metabolism dysregulation and may offer therapeutic potential for treating NASH.

**Supplementary Information:**

The online version contains supplementary material available at 10.1186/s43556-026-00409-4.

## Introduction

Nonalcoholic steatohepatitis (NASH) represents a severe stage of hepatic steatosis, which is mainly characterized by ballooning, inflammation, and even fibrosis [1, 2]. With disease advancement, NASH may progress into cirrhosis, hepatocellular carcinoma, and other end-stage liver diseases [3]. The global prevalence of NASH is currently 2%–6% and is estimated to surge up to 56% by 2030 [4]. However, few drugs have been approved for NASH treatment and elucidating its pathogenesis to develop effective treatments remains a major unmet basic and clinical need [5].

Hydrogen peroxide-inducible clone 5 (Hic-5), also known as transforming growth factor beta-1-induced transcript 1 (Tgfb1i1), was originally identified as a gene induced by hydrogen peroxide (H₂O₂) [6]. As a LIM-domain focal adhesion scaffold protein, Hic-5 plays a key role in promoting tumor extracellular matrix (ECM) deposition and remodeling. For example, Hic-5 promotes fibrillar adhesion formation through interaction with tensin1 and facilitates breast tumor cell growth and metastasis [7]. Hic-5 enhances cell-ECM mechanosignaling and associated actin cytoskeletal remodeling. This promotes myocardin-related transcription factor A (MRTF-A) nuclear translocation, thereby underscoring Hic-5's role in focal adhesion mechanobiology, stromal ECM remodeling, and tumor cell invasion.[8] Moreover, Hic-5 functions as a transcriptional coregulator for several genes. Previous evidence has suggested that coregulator appear to modulate the assembly/disassembly of the regulatory complex. As a coregulator, Hic-5 facilitates the assembly of transcription complexes on some glucocorticoid receptor target genes [9]. Besides, Hic-5 acts as both a positive and negative regulator of androgen receptor transcriptional activity, thereby influencing androgen-mediated regulation of cell growth, adhesion, and motility [10]. Similarly, Hic-5 participates in the transcriptional activation of the *c-fos* gene by serving as a scaffold within transcriptional complexes [11]. As for the function of Hic-5 in the liver. Our previous study has revealed that Hic-5 was highly expressed in hepatic stellate cells (HSCs), but neither liver sinusoidal endothelial cells, including endothelial cells of the liver central vein, nor Kupffer cells expressed Hic-5 [12]. Further research determined Hic-5 as a novel regulator of HSCs activation. However, the role of Hic-5 in hepatic lipid metabolism and NASH development remains unknown.

Lipid metabolism dysregulation in hepatocytes is crucial in NASH progression [13], and this process involves numerous physiological changes and mechanisms. PGE2 is an important inflammatory lipid mediator, facilitating intercellular communication across various physiological processes and contributing to fibrosis and metabolic disorders [14, 15]. Previous studies have indicated that during NASH progression, macrophages-derived PGE2 act on EP4 receptors of HSCs membrane, promoting HSCs activation and extracellular matrix deposition, thereby accelerating fibrosis [15]. In regulating hepatic lipid metabolism, PGE₂ has been shown to modulate cholesterol metabolism by activating the PKA/HNF4α/CYP7A1 pathway through hepatocyte EP3 receptors [16]. Collectively, these findings underscore the significant role of PGE₂ in liver pathophysiology. However, the relationship between Hic-5 and PGE2, and the role of PGE2 in regulating hepatic fatty acid metabolism, as well as targeted receptor is unclear.

In the present study, we found that Hic-5 contributes to fatty acid metabolism dysregulation in NASH, and this function is realized by its interaction with PTEN as a scaffold protein rather than a transcription coregulator. The N-terminal of Hic-5 binds c-Src and promots phosphorylation and inactivation of PTEN, which is bound to the C-terminal. This leads to increased phosphorylation of SP1, thus promoting PGE2 secretion and mediating hepatocellular fatty acid metabolism dysregulation. PGE2-EP4 axis mediates the regulation of Hic-5 on hepatocellular fatty acid synthesis. Our study not only uncovered the role of Hic-5 and PGE2 on fatty acid synthesis, but also elucidates the mechanistic link between them that drives NASH pathogenesis. This discovery establishes a novel link between HSCs and hepatocytes, and provided potential therapeutic target for NASH.

## Results

### Hic-5 is upregulated in patients with NASH and mouse models of NASH.

**Fig. 1:**
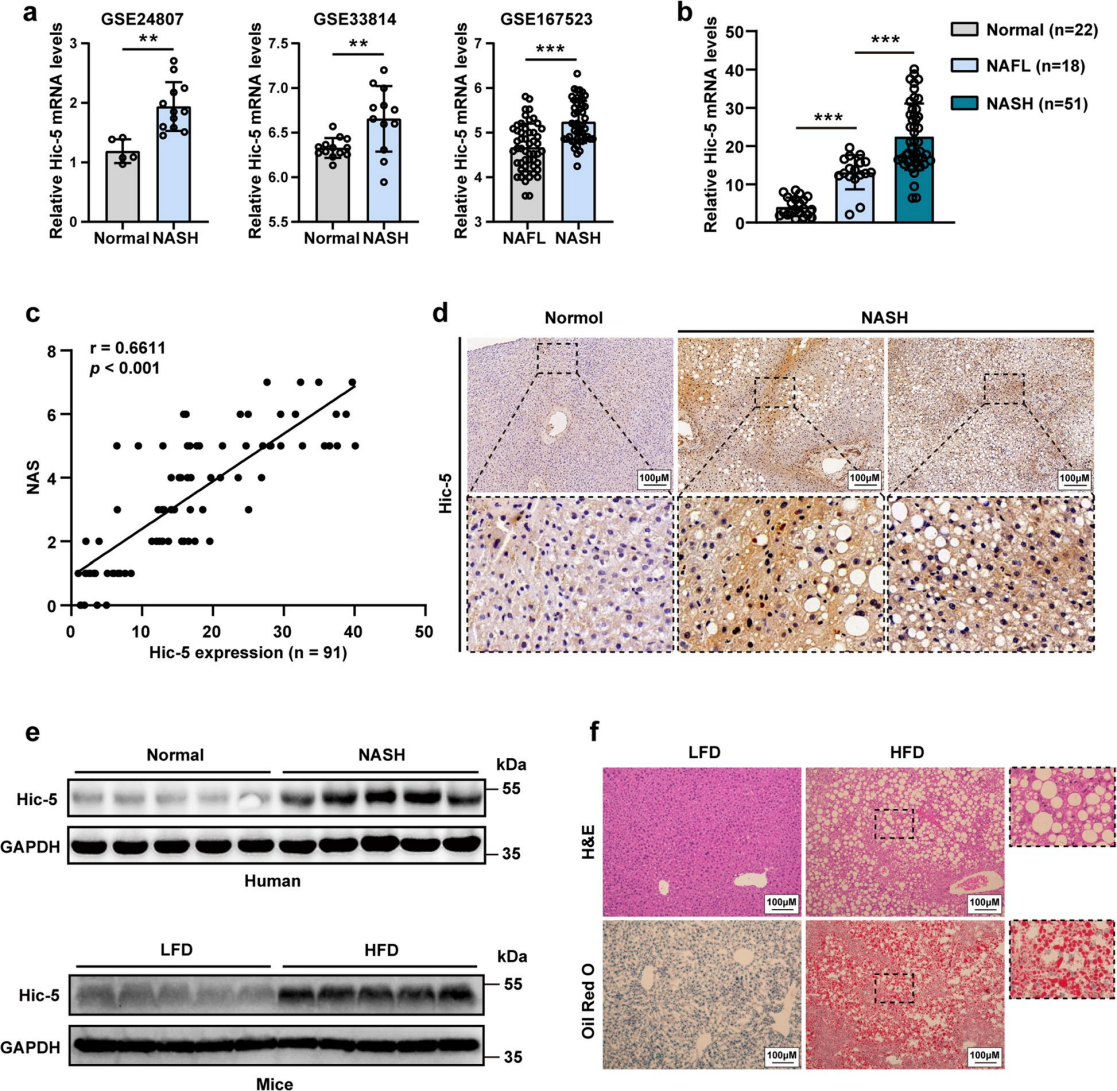
Hic-5 is upregulated in patients with NASH and mouse models of NASH. **a** The mRNA expression levels of Hic-5 in human transcriptome datasets (GSE24807, GSE33814 and GSE33814). **b** The mRNA expression levels of Hic-5 in liver tissues from patients with NASH (*n* = 51), patients with NAFL (n = 18) and normal controls (n = 22). **c** Correlation analysis between the expression of Hic-5 and NAS score. **d** Representative IHC of Hic-5 in the liver tissues from patients with NASH and normal controls. **e** The protein levels of Hic-5 in the liver tissues of patients with NASH and mouse models of NASH were examined by WB. **f** Representative H&E and Oil red O staining images in the liver tissues from mice fed with HFD or normal diet. Data are expressed as mean ± SD. *p < 0.05, **p < 0.01, ***p < *0.001*

We first investigated the expression of Hic-5 in three published human transcriptome datasets (GSE24807 and GSE33814 contain data from normal specimens and patients with NASH, and GSE167523 compares patients with NAFL and NASH), and two mice transcriptome datasets (GSE119340 and GSE35961 compares the expression of Hic-5 between high-fat diet (HFD) induced mouse models). The results indicated obvious elevation of Hic-5 in patients with NASH and increased with disease progression (Fig. 1a and Fig.S1a). We then collected human specimens, including 51 patients with NASH, 18 patients with non-alcoholic fatty liver (NAFL), and 22 normal controls. The histological manifestation of steatosis was revealed by hematoxylin and eosin (H&E) and Oil Red O staining (Fig. S1b), and the results of real-time polymerase chain reaction (RT-qPCR) indicated that the gene expression of *Hic-5* was upregulated in fatty livers and was gradually increased from NAFL to NASH (Fig. 1b). Furthermore, the degree of Hic-5 upregulation was positively correlated with NAS scores in human liver tissues (Fig. 1c). The upregulated Hic-5 protein level in human NASH tissues was confirmed by IHC and WB (Fig. 1d, e). Meanwhile, Hic-5 is highly expressed in HSCs in the liver of patients with NASH and co-localized with α-SMA according to single-cell RNA sequencing. (Fig. S1c). We further constructed mouse models of NASH by feeding with HFD diet for 24 weeks. H&E and Oil Red O staining indicated significant steatosis. Similarly, Hic-5 mRNA and protein levels increased in the liver of mouse models (Fig. 1f and Fig. S1d).

### Hic-5 deficiency alleviates hepatic lipid accumulation in experimental NASH models

**Fig. 2:**
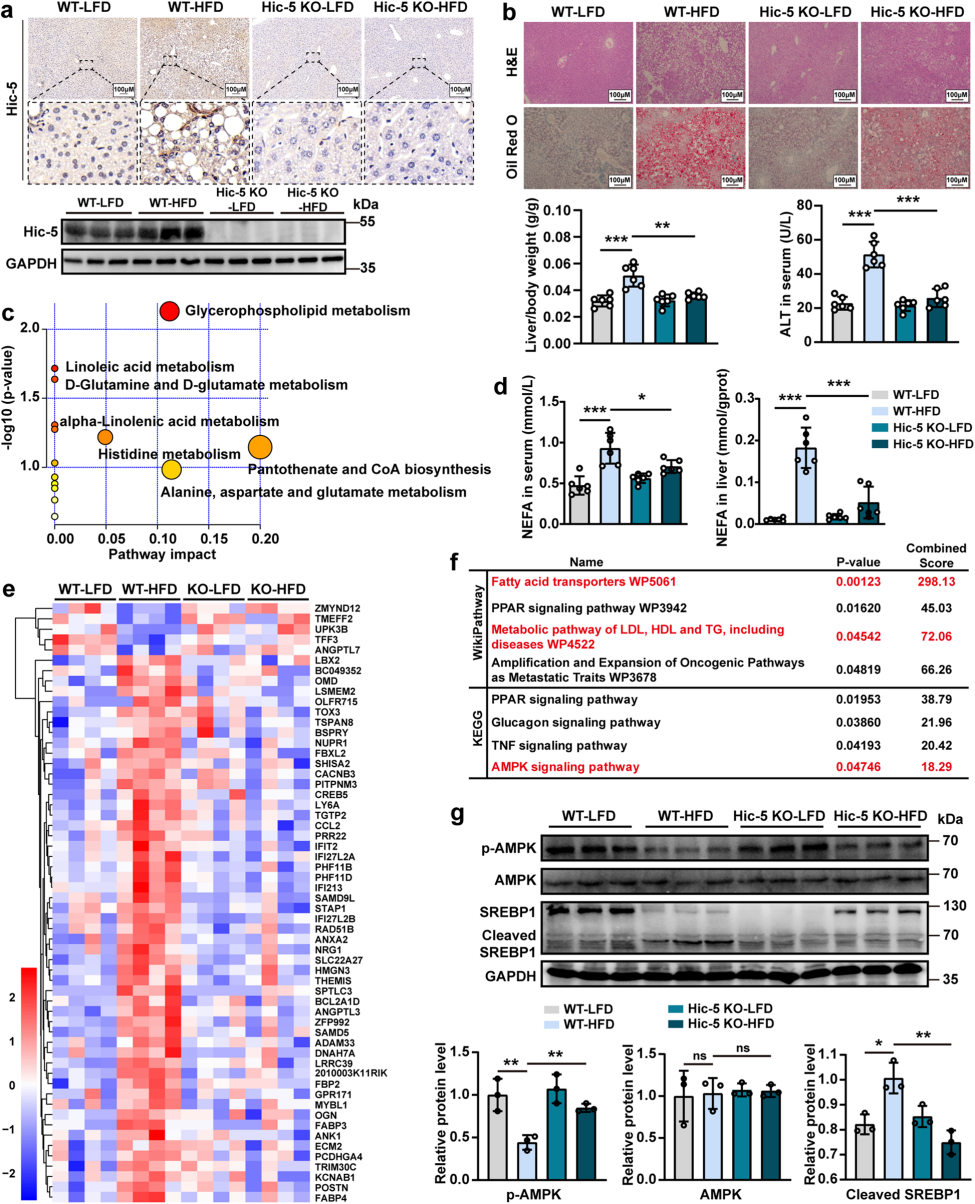
Hic-5 deficiency alleviates hepatic lipid accumulation in experimental NASH models. **a** IHC and WB demonstrated the successful knockout of Hic-5. **b** Representative H&E, Oil red O staining and liver/body weight (n = 6/group), serum ALT (n = 6/group) in Hic-5 KO and WT mice fed with HFD or LFD diet, the groups are WT-LFD, WT-HFD, Hic-5 KO-LFD, Hic-5 KO-HFD. **c** Metabolomics analysis on liver tissues (WT-LFD vs WT-HFD, WT-HFD vs Hic-5 KO-HFD). **d** Serum and liver tissue NEFA levels (*n* = 6/group). **e** RNA sequencing of the liver tissues (WT-LFD vs WT-HFD, WT-HFD vs Hic-5 KO-HFD). **f** WikiPathway and KEGG enrichment analysis (WT-LFD vs WT-HFD, WT-HFD vs Hic-5 KO-HFD). **g** The expression of p-AMPK and SREBP1 proteins in liver tissues were detected by WB (n = 3/group). Data are expressed as mean ± SD. *p < 0.05, **p < 0.01, ***p < *0.001*

To further confirm the role of Hic-5 in NASH, Hic-5 KO mice and littermate lacking the knockout were used to develop experimental NASH models by feeding with HFD or control diet (Fig. S2a). IHC and WB demonstrated the successful knockout of Hic-5 (Fig. 2a). H&E and Oil Red O staining of liver tissues revealed that Hic-5 deficiency improved histological morphology and alleviated steatosis compared with the model group (Fig. 2b). The liver/body weight was significantly decreased in Hic-5 KO mice fed with HFD diet compared to WT mice fed with the same diet, despite no significant change in body weight (Fig. 2b and Fig. S2b). Additionally, serum alanine transaminase (ALT), aspartate aminotransferase (AST), and inflammatory factors, such as interleukin 6 (IL-6), tumor necrosis factor-alpha (TNF-α), and interleukin 1β (IL-1β), were decreased, indicating less liver injury and inflammation (Fig. 2b and Fig. S2c, d). We then performed metabolomics. The enrichment analysis indicated that Hic-5 deficiency is associated with fatty acid metabolism pathways such as linoleic acid and α-linolenic acid (Fig. 2c). Subsequent serum and liver lipids assay further confirmed the results of metabolomics. Hic-5 deficiency reduces non-esterified fatty acids (NEFA) levels in HFD diet-fed mice (Fig. 2d), and trends in triglyceride (TG) level were consistent with NEFA (Fig. S2e).

Next, RNA sequencing was conducted to understand the effects of Hic-5 deficiency on the liver transcriptome. A total of 58 hub genes were identified (Fig. S3a-c). Based on the result of WikiPathway and KEGG enrichment analysis, Hic-5 deficiency is correlated with AMPK signaling pathway (Fig. 2e, f). The WB and IF of AMPK in liver tissues further demonstrated that HFD diet leads to decreased levels of AMPK phosphorylation, while Hic-5 deficiency promoted phosphorylation of p-AMPK (Fig. 2g and Fig. S3d). Additionally, sterol regulatory element-binding protein 1 (SREBP1), an important transcriptional regulator that regulates lipid synthesis, was significantly downregulated in its cleaved form in Hic-5 deficiency mice fed with HFD diet (Fig. 2g). IHC of FASN, another key enzyme of fatty acid synthesis, showed significant decrease (Fig. S3e). Fig. S4a-g further demonstrated the results of liver tissue metabolomics analysis.

### Hic-5 upregulation in hepatic stellate cells exacerbates hepatic lipid accumulation

**Fig. 3:**
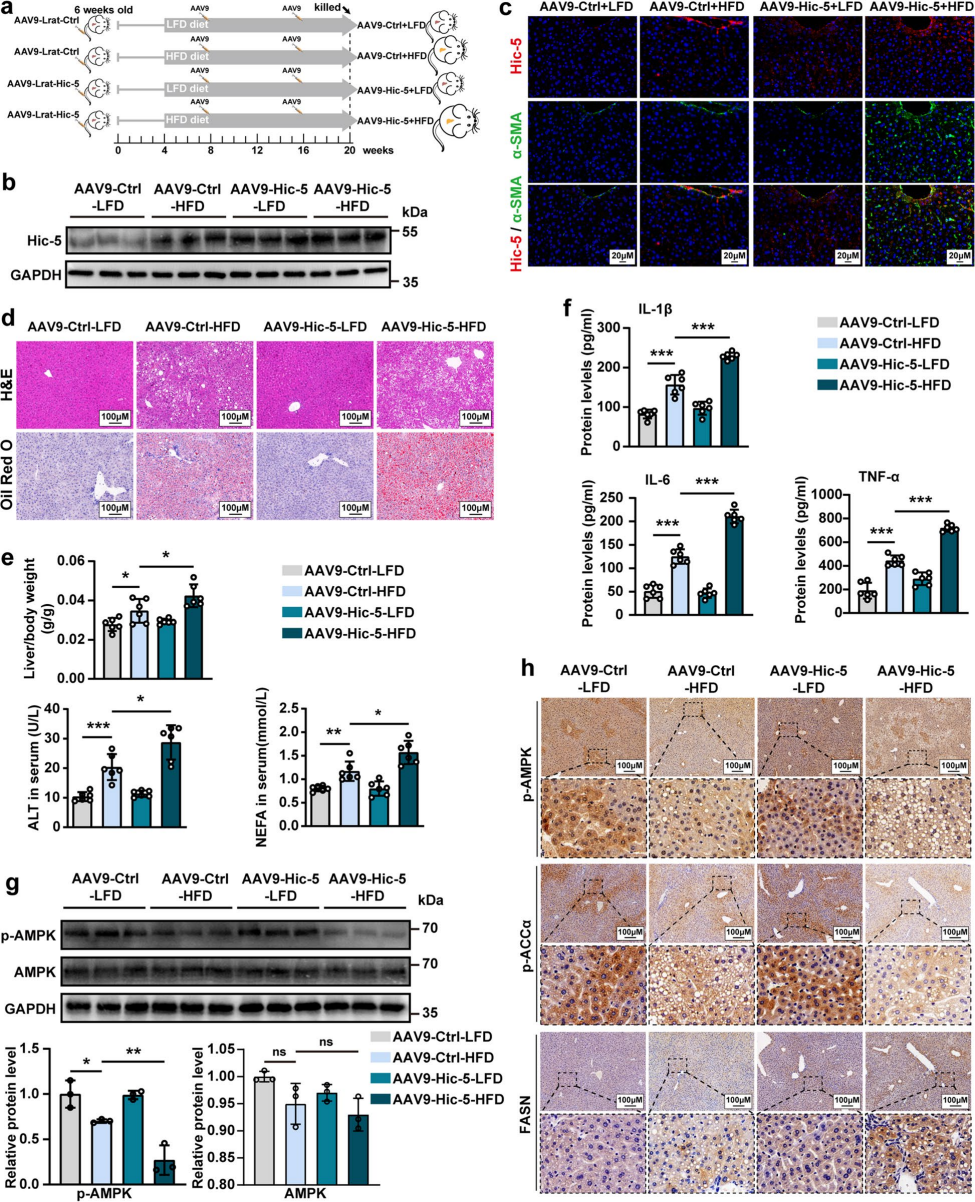
Hic-5 upregulation in hepatic stellate cells exacerbates hepatic lipid accumulation. **a** Schematic diagram of biulding Hic-5 HOE mice NASH models by treating with AAV9-Lrat-Hic-5 or AAV9-Lrat-null and feeding with HFD or normal diet, the groups are AAV9-Ctrl-LFD, AAV9-Ctrl-HFD, AAV9-Hic-5-LFD, AAV9-Hic-5-HFD. **b**, **c** WB and fluorescence colocalization of α-SMA and Hic-5 confirmed successful overexpression of Hic-5. **d** Representative H&E and Oil red O staining from liver tissues. (**e, f**) Liver/body weight (n = 6/group), serum ALT (n = 6/group), serum NEFA and inflammatory factor levels (n = 6/group). **g** The expression of Hic-5 and p-AMPK proteins in liver tissues were detected by WB (n = 3/group). **h** Representative IHC of p-AMPK, p-ACCα and FASN from liver tissues. Data are expressed as mean ± SD. *p < 0.05, **p < 0.01, ***p < *0.001*

As Hic-5 is highly expressed in HSCs, we specifically overexpressed Hic-5 in HSCs (Hic-5 HOE) by tail vein injection using adeno-associated virus 9 and the stellate cell-specific promoter Lrat and constructed mouse models of NASH by administering HFD diet (Fig. 3a). WB and fluorescence colocalization of α-SMA and Hic-5 confirmed successful overexpression (Fig. 3b, c). Histopathology of liver tissues and enzyme-linked immunosorbent assay demonstrated that HFD diet-fed Hic-5 HOE mice had worsened NASH phenotype with deeper hepatic steatosis, higher liver/body, increased liver injury and inflammatory factor secretion compared to HFD diet-fed WT mice (Fig. 3d-F and Fig. S5a). Interestingly, the change of overall body weight was not significant (Fig. S5b). However, we observed some degree of fibrosis occurred in HFD diet-fed Hic-5 HOE mice, but it remained relatively mild (Fig. S5c). This phenomenon indicates that the HFD diet we adopted may not contribute to the fibrosis process. Additionally, IHC and WB showed decreased phosphorylation of AMPK, accompanied by a decrease in phosphorylated ACCα (Fig. 3g, h). Conversely, FASN were increased according to the results of IHC (Fig. 3h). Altogether, these results indicate that Hic-5 overexpression in HSCs exacerbates hepatic lipid accumulation and affects the AMPK phosphorylation metabolic pathway.

### Hic-5 in hepatic stellate cells promotes hepatocellular fatty acid synthesis

**Fig. 4:**
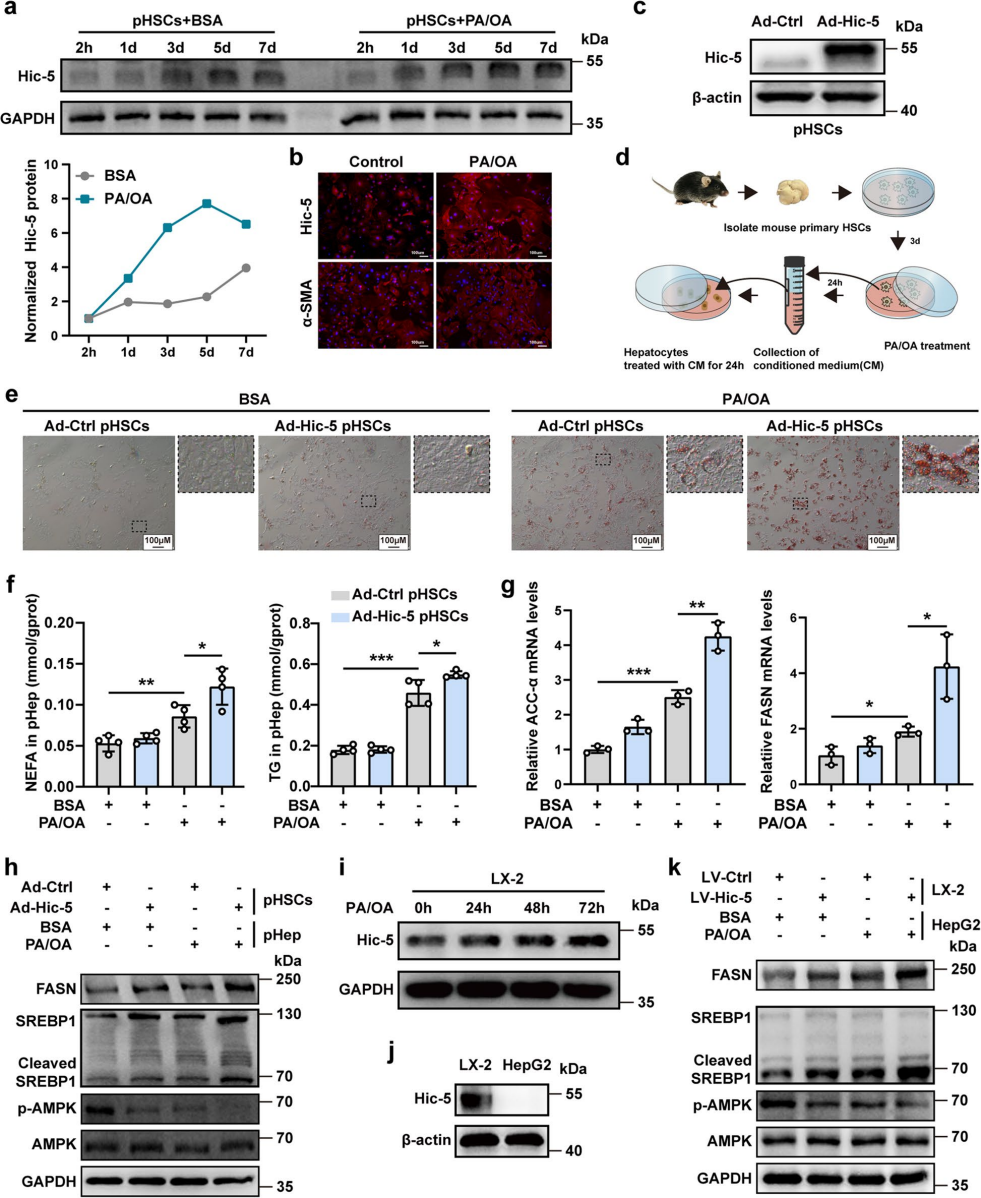
Hic-5 in hepatic stellate cells promotes hepatocellular fatty acid synthesis. **a** The protein expression of Hic-5 in pHSCs upon self-activation and PA/OA induction was detected by WB. **b** The fluorescence staining of Hic-5 and α-SMA in pHSCs after 3 days of isolation and exposed to PA/OA for 24 h. **c** Successful overexpression of Hic-5 in pHSCs using Ad-Hic-5 was confirmed by WB. **d** Schematic diagram of co-culture system of pHSCs and pHep, the groups are Ad-Ctrl pHSCs-BSA, Ad-Ctrl pHSCs-PA/OA, Ad-Hic-5 pHSCs-BSA, Ad-Hic-5 pHSCs-PA/OA. **e** Representative Oil red O staining of pHep after co-cultured with pHSCs overexpressing Hic-5. **f** The NEFA and TG levels in pHep after co-cultured with Ad-Hic-5 pHSCs (n = 4/group). **g** The mRNA expression levels of ACCα and FASN in pHep (n = 3/group). **h** The expression of p-AMPK, SREBP1 and FASN proteins in pHep after co-cultured with Ad-Hic-5 pHSCs were detected by WB. **i** The expression of Hic-5 in LX2 cells upon PA/OA induction was detected by WB. **j** The non-expression of Hic-5 in HepG2 was confirmed by WB. **k** The expression of p-AMPK, SREBP1 and FASN proteins in HepG2 cells after co-cultured with LV-Hic-5 LX-2 were detected by WB. Data are expressed as mean ± SD. *p < 0.05, **p < 0.01, ***p < *0.001*

Next, we developed a co-culture system of HSCs and hepatocytes to understand why Hic-5 upregulation promotes hepatocellular lipid accumulation. Firstly, we simulated an in vitro high-fat environment with palmitic acids and oleic acids (PA/OA), BSA as control, and cultured mouse primary HSCs (pHSCs) and the human HSC line LX-2. The expression of Hic-5 was upregulated in pHSCs with prolonged PA/OA induction (Fig. 4a), which was consistent with the trend of expression in liver tissues of patients and mouse models. Generally, pHSCs are self-activated in vitro. In order to exclude the influence of self-activation on the expression of Hic-5, we compared the activation of pHSCs at five different time points, including 0, 1, 3, 5, and 7 days with the exposure to PA/OA at the same time. It is seemed that the upregulation of Hic-5 in pHSCs, which were cultured with PA/OA, was stronger than the expression caused by its activation (Fig. 4a). Hic-5 and α-SMA fluorescence staining of pHSCs showed the same results (Fig. 4b). Therefore, we selected pHSCs after 3 days of isolation for the following experiments.

We then used adenoviruses (Ad-Hic-5 and Ad-Ctrl) to overexpress Hic-5 in pHSCs (Fig. 4c). Co-culture of pHSCs and primary hepatocytes (pHep) were developed (Fig. 4d). The conditioned media from PA/OA-exposed Ad-Hic-5 pHSCs markedly promoted lipid accumulation in pHep (Fig. 4e, f). Next, we analyzed the mRNA expression of *ACCα* and *FASN*, which are key enzymes for fatty acid synthesis in pHep. The results showed that *ACCα* and *FASN* were significantly upregulated in the Hic-5 overexpression co-culture system (Fig. 4g). Meanwhile, Hic-5 overexpression inhibited AMPK phosphorylation and promoted FASN upregulation in pHep. The expression of cleaved SREBP1 was upregulated, indicating increased fatty acid synthesis, despite some differences in the total protein expression of SREBP1 (Fig. 4h). We further validated this in cell lines. The expression of Hic-5 was upregulated in LX-2 cells with prolonged PA/OA induction (Fig. 4i). Next, lentiviruses (LV-Hic-5 and LV- Ctrl) were used to overexpress Hic-5 in LX-2 (Fig. S6a). Co-culture of LX-2 and HepG2 were developed (Fig. 4j and Fig. S6b). Hic-5 overexpression promoted lipid accumulation in HepG2 (Fig. S6c, d), and the expression of proteins mirrored that in primary cells (Fig. 4k). Furthermore, we knocked down Hic-5 in LX-2 using siRNA (Fig. S6e) and co-cultured with HepG2. The results revealed that inhibition of Hic-5 attenuated lipid accumulation in HepG2 (Fig. S6e). Altogether, these results indicate that Hic-5 in HSCs promotes hepatocellular lipid accumulation by increasing fatty acid synthesis.

### Hic-5 promotes PEG2 expression and secretion through SP1 in hepatic stellate cells

**Fig. 5:**
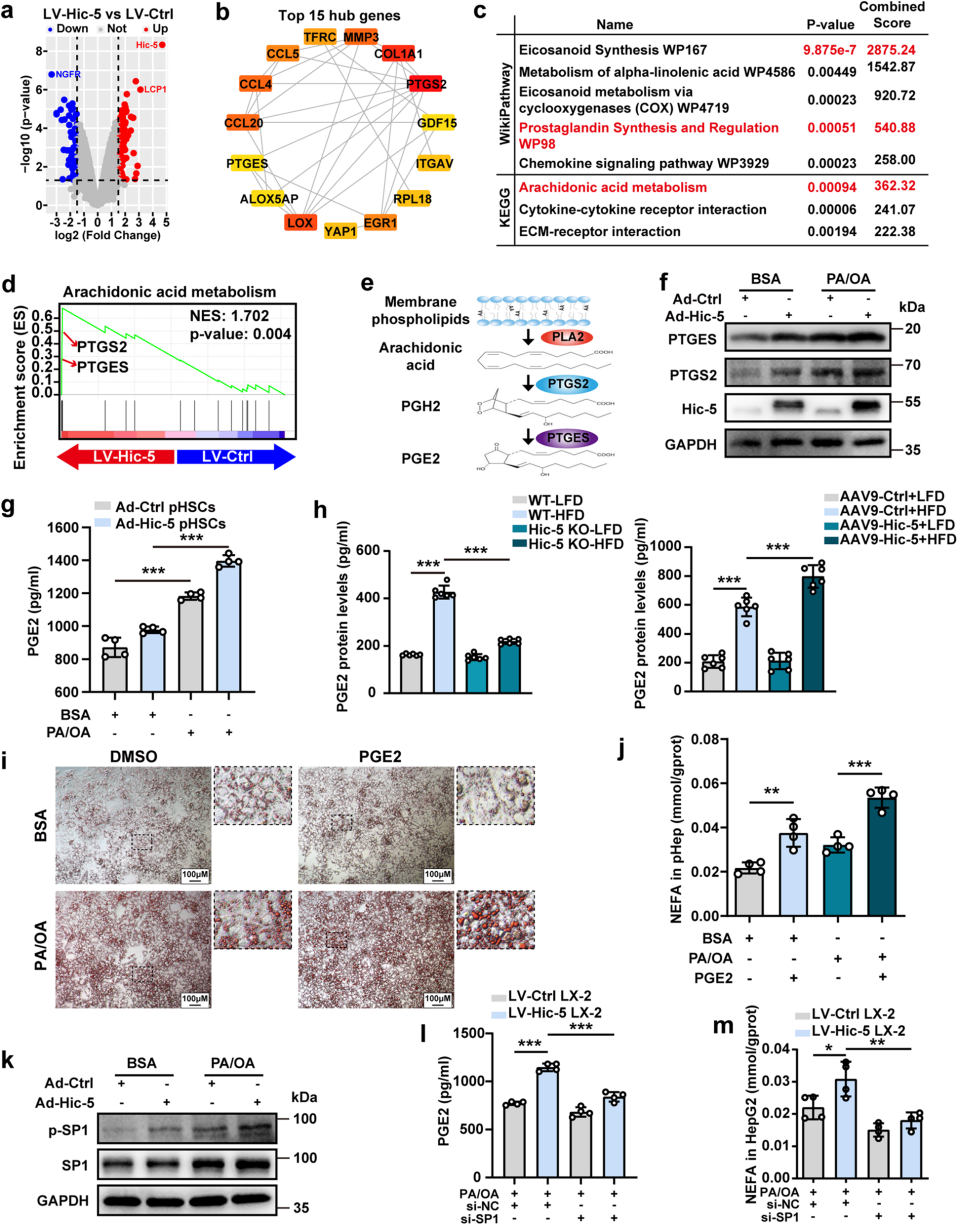
Hic-5 promotes PEG2 expression and secretion through SP1 in hepatic stellate cells. **a**, **b** The differential gene expression analysis and PPI network analysis of LX-2 cells (LV-Hic-5 vs LV-Ctrl). (**c**) The WikiPathway and KEGG enrichment analysis (LV-Hic-5 vs LV-Ctrl). **d** The GSEA analysis (LV-Hic-5 vs LV-Ctrl). **e** Schematic diagram of the PGE2 synthesis pathway. **f** The expression of PTGES and PTGS2 in Ad-Hic-5 pHSCs exposed to PA/OA or not. **g** The PGE2 levels from supernatant of pHSCs (n = 4/group). **h** Serum PGE2 levels in Hic-5 KO, Hic-5 HOE and WT mice fed with HFD or LFD diet (n = 6/group). **i** Oil red O staining of pHep treated with PGE2. **j** The NEFA levels in pHep treated with PGE2 (n = 4/group). **k** The expression of SP1 and p-SP1 in Ad-Hic-5 pHSCs exposed to PA/OA or not. **l** The PGE2 levels in the supernatant of LV-Hic-5 LX-2 cells after knockdown of SP1 (n = 4/group). **m** The NEFA levels in HepG2 were detected after knockdown of SP1 in LV-Hic-5 LX-2 cells and co-cultured with HepG2 (*n* = 4/group). Data are expressed as mean ± SD. *p < 0.05, **p < 0.01, ***p < *0.001*

To investigate the underlying mechanism of cross-talk between hepatic stellate cells and hepatocytes, we performed transcriptomic analysis of LX-2 cells which overexpressed Hic-5. Through differential gene expression analysis (log2FC > 1.5, P < 0.05), the total number of DEGs was found to be 187 in the LV-Hic-5 group, of which 112 were up-regulated and 75 were down-regulated (Fig. 5a). The top 15 highly connected genes were found by PPI network analysis (Fig. 5b). Based on the result of WikiPathway and KEGG enrichment analysis, we discovered that 15 hub genes were clustered in the arachidonic acid metabolism (Fig. 5c). GSEA analysis revealed that Hic-5 overexpression is positive correlation with arachidonic acid metabolism especially the expression of prostaglandin-endoperoxide synthase 2 (PTGS2) and prostaglandin E synthase (PTGES) (Fig. 5d). Prostaglandin E2 (PGE2) is a key bioactive metabolite of arachidonic acid with diverse biological functions. Phospholipase A2 (PLA2) catalyzes membrane phospholipids to release arachidonic acid, and the latter is catalyzed by PTGS2 and PTGES to produce PGE2 (Fig. 5e). The detection of protein expression in pHSCs and LX-2 revealed that Hic-5 overexpression upregulated PTGS2 and PTGES and was more pronounced when induced by PA/OA. (Fig. 5f and Fig. S7a). PGE2 assay determined that Hic-5 increased PGE2 secretion from pHSCs and LX-2 (Fig. 5g and Fig. S7b). Strikingly, HFD diet-fed Hic-5 deficiency mice presented lower serum PGE2 levels compared with HFD diet-fed WT mice (Fig. 5h). Conversely, Hic-5 HOE demonstrated increased serum PGE2 levels (Fig. 5h). We then used recombinant PGE2 to evaluate its effects on lipid accumulation in hepatocytes. Oil red O staining and lipids assays revealed that recombinant PGE2 promoted lipid aggregation in pHep and HepG2 (Fig. 5i, j and Fig. S7c, d). However, the PGE2 neutralizing antibody 2B5 inhibited this phenomenon. (Fig. S7e, f).

Then, we examined the protein level of SP1, which is a transcription factor that promotes PEG2 synthase expression. The results revealed that overexpressed Hic-5 in pHSCs and LX-2 upregulated SP1 and phosphorylated SP1, and was more pronounced when exposed to PA/OA (Fig. 5k and Fig. S8a). We then used siRNA to knock down SP1 in LV-Hic-5 LX-2 cells and found that Hic-5 overexpression-upregulated PTGS2 and PTGES were attenuated upon SP1 inhibition, with a subsequent decrease in PGE2 secretion (Fig. 5l and Fig. S8b). Co-culture of LV-Hic-5 LX-2 and HepG2 revealed that SP1 knockdown in LX-2 attenuated lipid accumulation in HepG2 (Fig. 5m and Fig. S8c, d). Additionally, increased AMPK phosphorylation as well as inhibition of FASN and cleaved SREBP1 in HepG2 was observed (Fig. S8e). Altogether, these results indicate that Hic-5 in HSCs promotes PGE2 expression and secretion through SP1, thereby disrupting fatty acid metabolism in hepatocytes.

### Hic-5 binds to c-Src in hepatic stellate cells promotes PTEN phosphorylation and activates SP1 activity

**Fig. 6:**
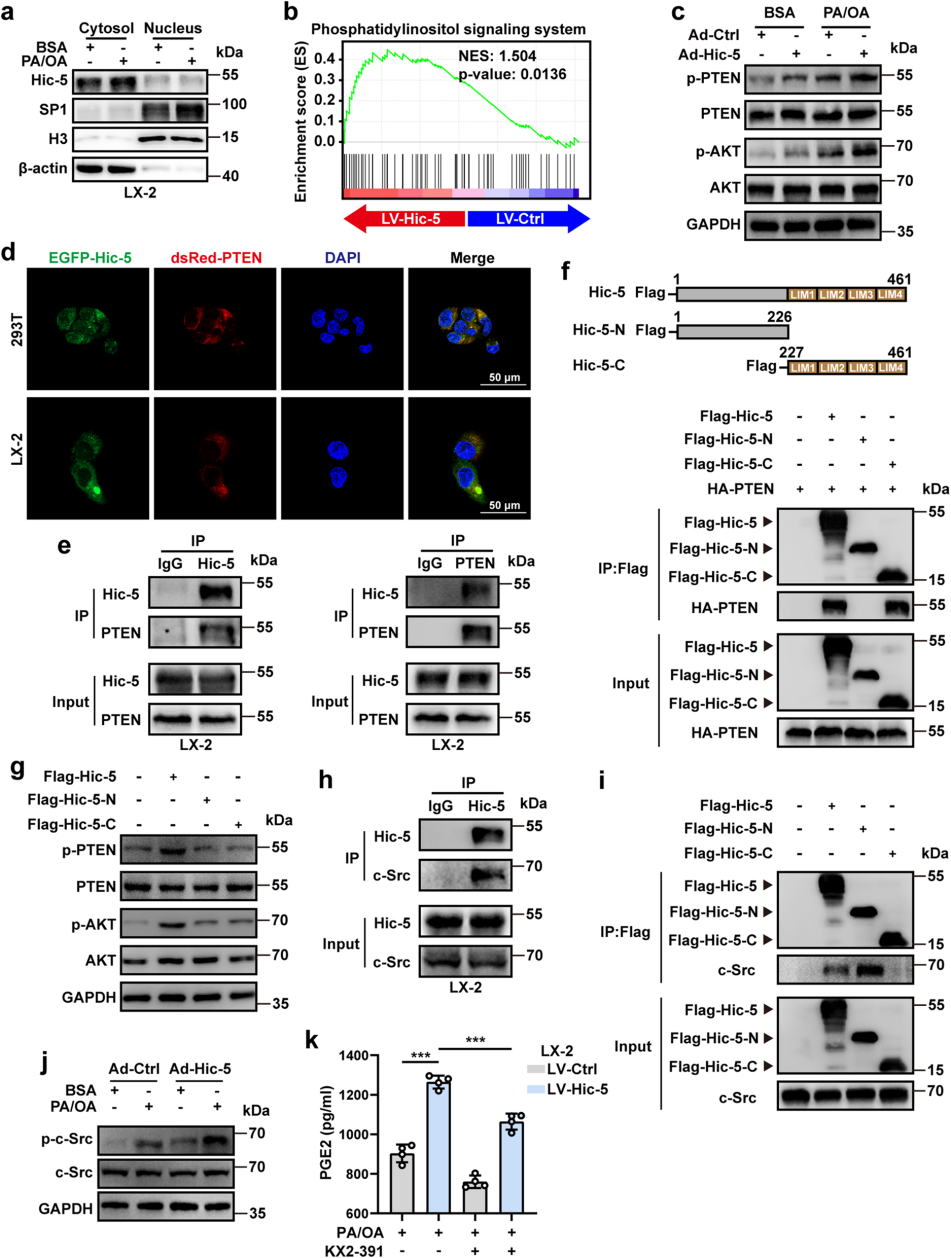
Hic-5 binds to c-Src in hepatic stellate cells promotes PTEN phosphorylation and activates SP1 activity. **a** The expression of Hic-5 and SP1 in LX-2 were detected according to nucleoplasmic separation. **b** The GSEA analysis (LV-Hic-5 vs LV-Ctrl). **c** Phosphorylation of AKT and PTEN in Hic-5 overexpressed pHSCs were detected by WB. **d** Immunofluorescence colocalization of Hic-5 and PTEN. **e** Co-IP results of Hic-5 and PTEN in LX-2 cells. **f** FLAG-tagged antibody labels the full-length and N-terminal of Hic-5, HA-tagged antibody labels the C-terminal of Hic-5, and Co-IP results of Hic-5 domains and PTEN in 293 T cells. **g** Phosphorylation of AKT and PTEN when overexpressed the full-length, N-terminal and C-terminal of Hic-5 in 293 T cells were detected by WB. **h** Co-IP results of Hic-5 and c-Src in LX-2 cells. **i** Co-IP results of Hic-5 domains and c-Src in 293 T cells. **j** Phosphorylation of c-Src in Hic-5 overexpressed pHSCs were detected by WB. **k** The PGE2 levels in the supernatant of LV-Hic-5 LX-2 cells when treated with s-Src inhibitor KX2-391

Whether Hic-5 directly targets SP1 to upregulate its expression is unclear. Therefore, we performed nucleoplasmic separation of LX-2 which was exposed to PA/OA and BSA. Interestingly, Hic-5 was overwhelmingly expressed in the cytoplasm, whereas SP1 entry into the nucleus was evident and was more pronounced in the nucleus when exposed to PA/OA (Fig. 6a). The distribution of Hic-5 and SP1 in the nucleus and cytoplasm indicated that there is no direct regulatory relationship between Hic-5 and SP1, and some unknown molecules may be involved. Enrichment analysis showed a positive correlation between the PI3K/AKT signaling pathway and Hic-5 in LX-2 cells (Fig. 6b). Elevated AKT phosphorylation after Hic-5 overexpression was observed and was aggravated by PA/OA induction. The phosphorylation level of phosphatase and tensin homolog (PTEN), a negative AKT regulator, was also increased (Fig. 6c and Fig. S9a). Immunofluorescence colocalization showed that Hic-5 and PTEN co-localized in the cytoplasm both in LX-2 and 293 T cells (Fig. 6d). We further performed Co-IP and found that Hic-5 could directly bind to PTEN (Fig. 6e). Next, we constructed FLGA-labeled Hic-5 and HA-labeled PTEN plasmids and transfected 293 T cells. The same results were observed (Fig. S9b).

The C-terminal region of Hic-5 contains four LIM domains, which are the main structural domains that bind to proteins and fulfill their functions. To confirm the binding of PTEN to the C-terminal of Hic-5, we constructed separate C-terminal, N-terminal, and full-length plasmids (Fig. 6f). Co-IP experiments were performed after transfection of these plasmids into 293 T cells. Unsurprisingly, the results indicated that PTEN binds directly to the C-terminal of Hic-5 (Fig. 6f). However, the phosphorylation levels of PTEN and AKT were reduced when transfected with the C-terminal alone (Fig. 6g), which was contrary to the results of Hic-5 overexpression in LX-2 and 293 T cells. Perhaps the combination of the C-terminal and PTEN is not sufficiency to upregulate the phosphorylation of the latter, and the N-terminal of Hic-5 may bind to other proteins and promote PTEN phosphorylation. We continued to search for possible binding proteins. c-Src, a kinase that phosphorylates PTEN, was found to bind to Hic-5 (Fig. 6h). Moreover, the combination of N-terminal and c-Src was detected in 293 T cells (Fig. 6i). Then we performed verification in LX-2 cells. When Hic-5 was transfected, the phosphorylation levels of PTEN and AKT significantly increased, and PGE2 secretion also increased. However, when the N-terminal or C-terminal was transfected separately, the phosphorylation of PTEN and AKT, as well as the secretion of PGE2, were significantly inhibited (Fig. S9c, d). Besides, the expression of c-Src in pHSCs and LX-2 was similar with PTEN (Fig. 6j and Fig. S9e), and s-Scr inhibitor KX2-391 significantly reduced PGE2 secretion (Fig. 6k). The above findings indicate that Hic-5 can't bind to c-Src in the absence of the N-terminal domain, preventing PTEN from phosphorylated. Meanwhile, in the absence of the C-terminal domain, since Hic-5 fails to bind to PTEN, the regulatory effect of c-Src on PTEN is diminished even when c-Src is present. Subsequently, we isolated pHSCs from Hic-5 KO mice for in vitro high-fat intervention, which yielded opposite trends in downstream protein expression and PGE2 secretion compared to Hic-5 overexpression (Fig. S9f, g).

### Hic-5 promotes hepatocellular fatty acid synthesis through the PGE2-EP4 axis

**Fig. 7:**
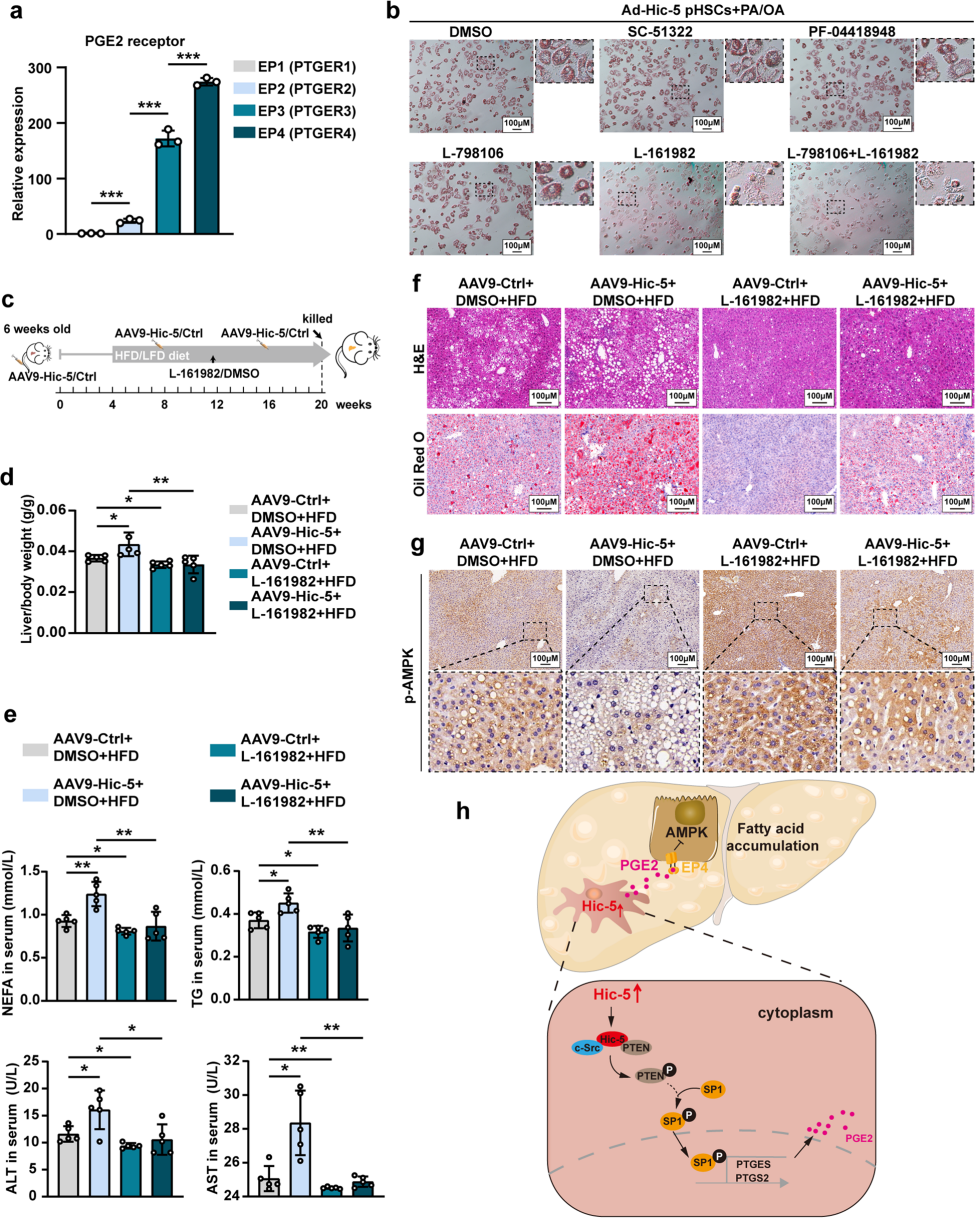
Hic-5 promotes hepatocellular fatty acid synthesis through the PGE2-EP4 axis. **a** The mRNA expression levels of EP1, EP2, EP3 and EP4 in HepG2 after co-cultured with LV-Hic-5 LX-2 (n = 3/group). **b** Representative Oil red O staining of pHep when PGE2 receptor inhibitors exist and cultured with conditioned media from PA/OA-exposed Ad-Hic-5 pHSCs. **c** Schematic diagram of treating HFD-fed Hic-5 HOE mice with EP4 inhibitor L-161982, the groups are AAV9-Ctrl + DMSO + HFD, AAV9-Hic-5 + DMSO + HFD, AAV9-Ctrl + L-161982 + HFD, AAV9-Hic-5 + L-161982 + HFD. **d**, **e** Liver/body weight of mice (n = 5/group), Serum NEFA, TG, ALT and AST (n = 5/group) levels. **f**, **g** Representative H&E and IHC of p-AMPK, P-ACCα and FASN from liver tissues. **h** The mechanism diagram of Hic-5 in regulating hepatocellular fatty acid synthesis. Data are expressed as mean ± SD. *p < 0.05, **p < 0.01, ***p < *0.001*

PGE2 is extensively involved in cellular metabolic processes by acting on four receptors, including EP1, EP2, EP3, and EP4. We found EP4 receptor was most significantly upregulated in HepG2 when co-cultured with LV-Hic-5 LX-2 through RT-qPCR (Fig. 7a). The inhibitor of four receptors SC-51322, PF-04418948, L-798106 and L-161982 were used to identify the involved receptor in Hic-5 regulation on hepatocellular fatty acid metabolism. Oil red O staining showed that lipid droplet accumulation was minimal in the presence of EP4 receptor inhibitor. The inhibitory effects of EP1, EP2, and EP3 were not significant, while the combined presence of EP3 and EP4 resulted in an inhibitory effect similar to that of EP4 alone. (Fig. 7b). We then used corresponding siRNA to knockdown PGE2 receptors respectively in HepG2 cells (Fig. S10a) and co-cultured them with LV-Hic-5 LX-2. Likewise, EP4 receptor knockdown attenuated lipid droplet accumulation most significantly and demonstrated the lowest NEFA and TG levels compared to knockdown of EP1, EP2, and EP3 receptors (Fig. S10b, c). Furthermore, the inhibition of EP4 in HepG2 cells showed the same Oil red O staining results (Fig. S10c).

We intraperitoneally injected EP4 receptor inhibitors to HFD diet-fed WT and Hic-5 HOE to confirm whether EP4 receptor inhibition alleviates the NASH phenotype exacerbated by Hic-5 overexpression (Fig. 7c). H&E and Oil Red O staining revealed that EP4 receptor inhibition alleviated Hic-5 overexpression-induced steatosis, and HFD diet-fed Hic-5 HOE mice exhibited lower liver/body weight, decreased liver injury when intraperitoneally injected L-161982 (Fig. 7d-f). IHC of liver tissues showed that Hic-5 HOE-induced downregulation of phosphorylated AMPK and phosphorylated ACCα, as well as upregulation of FASN were reversed by L-161982 treatment (Fig. 7g and Fig. S10d). The unexpected bonus was that EP4 receptor inhibition still had a palliative effect on fibrosis, although the fibrosis caused by Hic-5 HOE was mild (Fig. S10e). Briefly, HSCs Hic-5 binds to c-Src and PTEN to promote PGE2 secretion, leads to downregulation of AMPK phosphorylation and subsequently upregulation of FASN and SREBP1 by acting on hepatocyte EP4 receptors, and ultimately promotes hepatocellular fatty acid synthesis (Fig. 7h). Pharmacologic inhibition of EP4 reversed NASH phenotype exacerbation caused by Hic-5 overexpression in HSCs.

## Discussion

Disruption of hepatocellular lipid metabolism is a crucial event in the pathogenesis of NASH [17], with its regulatory mechanisms being intricate and poorly elucidated. A plethora of evidence suggests that hepatocellular lipid metabolism disorders are affected by intrahepatic cross-talk [18], and this process is modulated by liver microenvironmental cells such as macrophages, endothelial cells, and even immune cells. For example, macrophage XBP1 induces M1 macrophage transformation and pro-inflammatory cytokine secretion, promoting lipid accumulation in hepatocytes [19]. Neutrophil-derived EVs, enriched with miR-223/APOE, attenuate hepatic steatosis by suppressing lipid gene expression in hepatocytes [20]. Furthermore, WNT2, which is secreted by sinusoidal endothelial cells, controls cholesterol uptake and bile acid conjugation in hepatocytes through the receptor, FZD5 [21]. The effect of the cellular network between non-parenchymal cells and hepatocytes on hepatic lipid metabolism remains significant despite the small proportion of these non-parenchymal cells. Notably, HSCs, the liver's primary profibrotic drivers known for secreting extracellular matrix to promote fibrosis [22, 23], are also emerging as regulators of hepatocellular lipid metabolism. Studies have revealed that HSCs-derived free retinol during activation is absorbed by hepatocytes in a contact-dependent manner and mediates metabolic cross-talk between HSCs and hepatocytes [24]. Furthermore, experimental depletion of HSCs in murine NASH models significantly attenuates hepatic steatosis [25]. However, research in this area has largely remained phenotypic, with specific molecular targets and detailed regulatory mechanisms still elusive. Our previous research identified Hic-5 as a highly expressed gene in HSCs within the liver, serving as a key activator of HSCs [12]. Here, we discovered a significant upregulation of Hic-5 expression both in NASH patients and mouse models, while Hic-5 deficiency notably ameliorates hepatic steatosis in NASH mice, with the most significant improvement observed in the metabolism of fatty acids. Given that hepatocytes are the primary site of hepatic fatty acid metabolism,[26, 27] our finding led us to hypothesize that Hic-5 in HSCs may influence hepatocellular fatty acid metabolism through cross-talk. In this study, we demonstrated the role of Hic-5 in regulating lipid metabolism using systemic Hic-5 KO mice. Although Hic-5 is highly expressed in HSCs in the liver, the systemic KO of Hic-5 remains insufficiently convincing to fully rule out potential interference from Hic-5 in other liver cells. Subsequent adenovirus-mediated specifically overexpression of Hic-5 in HSCs resulted in an aggravated disease phenotype, and a follow-up primary cell co-culture system provided direct evidence of cross-talk. While these experiments partially addressed the limitations, the ideal in vivo evidence should be obtained from HSCs-Hic-5 specific KO mice. Therefore, in future studies, HSCs-Hic-5 specific KO mice are essential to refine the phenotypic observations and clarify the underlying mechanisms.

AMPK serves as a crucial regulatory factor in maintaining metabolic homeostasis, playing a pivotal role in fatty acid metabolism by phosphorylating various substrates [28, 29]. We observed a marked increase in phosphorylation levels upon Hic-5 deficiency at the histological level. SREBP1 and FASN, key enzymes in fatty acid synthesis [30, 31], are negatively regulated by phosphorylated AMPK [32, 33], exhibiting expression patterns opposite to those of AMPK. Changes in the AMPK signaling pathway were further validated through experiments involving mice with HSCs-specific overexpression of Hic-5 and co-cultures of cells, indicating that Hic-5 regulates fatty acid synthesis by influencing the AMPK signaling pathway within hepatocytes. In the present study, we found that Hic-5 upregulation was positively correlated with arachidonic acid metabolism, which is an important pathway for PGE2 synthesis [34], and was especially correlated with the expression of PGE2 synthases, including PTGES and PTGS2 [35]. Serum PGE2 levels were significantly decreased and elevated in the Hic-5 KO and Hic-5 HOE NASH mouse models respectively. In that case, serum PGE2 may serve as a potential biomarker for NASH progression, and its validation through large-scale clinical samples represents a necessary step for subsequent research. The upregulation of Hic-5 expression promotes PGE2 secretion in HSCs, while PGE2 neutralizing antibodies inhibit hepatocellular lipid accumulation. These results suggest that PGE2 may mediate the regulation of hepatocellular fatty acid metabolism by HSCs Hic-5. Nevertheless, our study has certain limitations. Although we have demonstrated at the cellular level that PGE2 derived from HSCs promotes fatty acid synthesis in hepatocytes, there is still a lack of some in vivo experimental evidence. Future research could involve using adenovirus carrying the HSCs-specific promoter Lrat to upregulate PGE2 secretion in HSCs-Hic-5 specific KO mice, thereby further validating its role in vivo. It is well-known that the function of PGE2 relies primarily on its binding to four different G protein-coupled receptors [36, 37]. Previous studies have demonstrated that the regulation of PGE2 on hepatocellular cholesterol metabolism is mediated by EP3 receptors [16]. However, in this study, inhibition of EP4 showed the most significant reduction of Hic-5-induced fatty acids accumulation Additionally, application of L-161982 at the histological level markedly reversed the deterioration of NASH phenotype induced by Hic-5 overexpression, which also offers potential drug and interventional directions for NASH treatment. Given the difficulty of targeting HSCs, developing drugs to inhibit EP4 receptors in hepatocytes may be a promising approach.

Mechanistically, Hic-5 promotes PGE2 secretion from HSCs through activation of the PTEN/AKT/SP1 signaling axis. SP1 is a known transcription factor for *PTGES* and *PTGS2*.[38, 39] In our study, Hic-5 overexpression led to an upregulation of total SP1 protein levels, along with increased phosphorylation at serine 739. Although Hic-5 can function as a transcriptional coregulator [9–11], the differential distribution of Hic-5 and SP1 observed in nucleoplasmic separation argues against a direct interaction in regulating SP1. It has been reported that AKT can promote nuclear translocation of phosphorylated SP1, exerting subsequent transcriptional regulatory effects [40, 41]. In our study, upregulation of Hic-5 increased the phosphorylation of both AKT and SP1. Phosphorylation at S739 is known to enhance SP1 protein stability,[42] which may contribute to the observed increase in total SP1 levels. PTEN, a negative regulator of AKT [43], exhibited increased phosphorylation in Hic-5 overexpressed pHSCs, and Hic-5 can directly bind to PTEN. As a focal adhesion scaffold LIM-containing protein, the LIM domain of Hic-5 is located at the C-terminal [44], precisely where PTEN binds to. Nevertheless, the binding at the C-terminal contradicts the trend of PTEN phosphorylation. c-Src has been reported to promote the apparent tyrosine phosphorylation of PTEN [45]. As verified by Co-IP, the N-terminal of Hic-5 could bind to c-Src, and inhibition of c-Src reduced PGE2 secretion. These results reveal the mechanism by which Hic-5 promotes PGE2 secretion in HSCs, acting as a focal adhesion scaffold protein rather than a transcription coregulator. The N-terminal of Hic-5 binds to c-Src, promoting phosphorylation and inactivation of C-terminal binding PTEN, leading to increased phosphorylation of AKT and SP1. SP1 then exerts its transcriptional regulatory effects on PTGES and PTGS2, promoting PGE2 secretion and mediating hepatocellular fatty acid metabolism disorder.

In this study, we investigated the regulatory role of Hic-5 in lipid metabolism. Overall, the upregulation of Hic-5 appears to function as one of the "engines" driving the progression of NASH. Hic-5 enables HSCs to sense metabolic stress and integrate signals, thereby regulating hepatocellular lipid metabolism through cross-talk. This becomes an indispensable factor in the early lipid metabolism disorder of NASH. As for the reason for the upregulation of Hic-5, it might be the result of the joint promotion of transcriptional activation and post-translational modification of proteins, which warrants further investigation. As metabolic disorders induce the gradual worsening of NASH, HSCs transition into a pro-fibrotic state, becoming central executors of fibrosis. At this stage, the function of Hic-5 may shift toward regulating the excessive accumulation of the extracellular matrix (ECM). At present, we only observed mild fibrosis following Hic-5 upregulation, which may be attributed to the dietary model and feeding duration employed. However, the role of Hic-5 in advanced stage of NASH should not be underestimated, and the c-Src/PTEN/PGE2 axis may be insufficient to regulate the overproduction of ECM. Therefore, extending HFD feeding duration or construct other diet-induced NASH fibrosis models to investigate the role of Hic-5 in the progression of NASH to cirrhosis should be taken into consideration. In summary, we discovered the regulation of hepatic lipid metabolism by HSCs and demonstrated the role of Hic-5 in NASH. Hic-5 aggravates the development of NASH by increasing PGE2 secretion and acting on hepatocellular receptor EP4, which promote fatty acid synthesis. Targeting Hic-5 may bring light to the treatment of NASH.

## Materials and methods

### Human liver samples

Human NASH liver specimens were collected from patients who underwent hepatectomy in the Affiliated Hospital of Southwest Medical University due to benign disease such as hepatic hemangioma, intrahepatic bile duct stones, and with independent assessment by two pathologists based on NAFLD activity scores (NAS). Hepatic steatosis due to hepatitis B virus, alcohol, etc. was excluded. Normal control liver specimens were obtained with absence of NASH. Informed consent of tissue collection was obtained from patients before surgery. Study of these specimens was approved by the Ethics Committee of Southwest Medical University (KY2022167) and was conducted in accordance with the 1975 Declaration of Helsinki.

### Animal models and sample collection

Hic-5 knockout (Hic-5 KO) mice based on a C57BL/6 J background were purchase from Cyagen Biosciences (Suzhou, China). Mice were housed in a temperature and humidity-controlled experimental animal room. After breeding, 6–8 weeks male Hic-5 KO mice were used to generate NASH model by feeding control or a high-fat diet (HFD) which contain 21.2% fat, 49.1% carbohydrate, 19.8% protein, and 0.2% cholesterol for 24 weeks (n = 8 per group). Littermate lacking the knockout were used as control. Wild type (WT) mice were purchase from Vital River Co., Ltd (Beijing, China), and hepatic stellate cell-specific Hic-5 overexpression (Hic-5 HOE) mice were generated by injecting adeno-associated virus 9 (AAV9)-lecithin retinol acyltransferase (Lrat)-null or AAV9-Lrat-Hic-5 (1 × 10^12^ vg/mice) into the tail vein. Mice were then fed control or HFD diet for 12 weeks (n = 8 per group). At the end of feeding, the serum and tissues were collected for subsequent experiments. All the animal experiments we performed followed the Animal Experimentation Ethics Committee of Southwest Medical University (20,211,119–047).

### Statistical analysis

Data were expressed as mean ± standard deviation and analyzed by the unpaired two-tailed Student’s t-test (the statistical significance of two groups) or one-way ANOVA analysis (the statistical significance of multiple groups). *P < 0.05, **P < 0.01, ***P < 0.001 was considered statistically significant. Statistical analysis was performed using GraphPad Prism Version 8.0.

## Supplementary Information

Supplementary Material 1.

## Data Availability

Authors declared that all and the other data supporting the findings of this study are available within the paper. The Bulk-RNA seq are presented in CNCB-NGDC database (HRA015688), and the raw data that support the findings of this study are available from the corresponding author upon reasonable request.
