# Supplementary material for: Hic-5 promotes the progression of nonalcoholic steatohepatitis by regulating hepatocellular fatty acid metabolism through the PTEN/PGE2/EP4 axis
Source: Mol Biomed. 2026 Feb 9;7:10. doi: 10.1186/s43556-026-00409-4 (PMC12886700; doi:10.1186/s43556-026-00409-4)
Supplement: Supplementary file 1 — Supplementary Material 1. [file 43556_2026_409_MOESM1_ESM.docx]

**Hic-5 promotes the progression of nonalcoholic steatohepatitis by regulating hepatocellular fatty acid metabolism through the PTEN/PGE2/EP4 axis**

Zhiwei Huang^1,2#^, Peng Tan^1,2#^, Boyuan Gu^1#^, Shenglu Liu^1^, Han Li^1^, Jiatong Chen^1^, Bingyu Ren^1^, Lei Sun^1^, Jian Wen^1,2^, Yu Li^3*^, Wenguang Fu^1,2*^

^1^ Department of General Surgery (Hepatobiliary Surgery), Department of Biliary-Pancreatic Center, The Affiliated Hospital, Southwest Medical University, Luzhou 646000, China.

^2^ Metabolic Hepatobiliary and Pancreatic Diseases Key Laboratory of Luzhou City, Academician (Expert) Workstation of Sichuan Province, The Affiliated Hospital, Southwest Medical University, Luzhou 646000, China.

^3^ CAS Key Laboratory of Nutrition, Metabolism and Food Safety, Shanghai Institute of Nutrition and Health, University of Chinese Academy of Sciences, Chinese Academy of Sciences, Shanghai 200031, China.

^#^These authors made equal contributions to this work.

*Correspondence: Wenguang Fu, Department of General Surgery (Hepatobiliary Surgery), Department of Biliary-Pancreatic Center, The Affiliated Hospital, Southwest Medical University, 25th Taiping Street, Luzhou 646000, Sichuan Province, China.

E-mail: [fuwg@swmu.edu.cn](mailto:fuwg@swmu.edu.cn)

Yu Li, CAS Key Laboratory of Nutrition, Metabolism and Food Safety, Shanghai Institute of Nutrition and Health, University of Chinese Academy of Sciences, Chinese Academy of Sciences, No. 320 Yueyang Road, Xuhui District, Shanghai 200031, China.

E-mail: [liyu@sinh.ac.cn](mailto:liyu@sinh.ac.cn)

**Supplementary materials and methods**

**Histopathology of liver**

The fresh liver tissues fixed in 4% paraformaldehyde and embedded in paraffin were used for further staining. For H&E staining, sections of 4µm were cut and sequentially stained with hematoxylin and eosin following the manufacturer´s instructions (G1120, Solarbio). Two pathologists evaluated H&E-stained liver sections individually based on NAFLD activity scores (NAS) according to the degree of steatosis, inflammation, and hepatocyte ballooning. For Sirius red staining, paraffin sections were dewaxed, hydrated and stained in Sirius red. Next, sections were dehydrated with anhydrous ethanol and cleaned in xylene, then sealed with neutral resin. The steps of dewaxing and hydration were applied to Masson's trichrome staining firstly. Then, sections were immersed in a solution of potassium dichromate at 65℃ for 30 minutes and rinsed with distilled water. Subsequent stains were performed sequentially using hematoxylin, ponceau, phosphomolybdic acid, and aniline blue. Finally, sections were differentiated using glacial acetic acid and dehydrated with anhydrous ethanol, then sealed with neutral resin. As for Oil Red O staining, fresh frozen sections were fixed and then immersed in oil red staining solution. Subsequently, the sections were rinsed with distilled water and stained with hematoxylin. Under microscope, the lipid droplets are presented to be reddish-orange and the nucleus are blue.

**Immunohistochemistry and immunofluorescence staining**

Immunohistochemistry of liver tissues were performed by using anti-Hic-5 (10565-1-AP, Proteintech), anti-p-AMPK Thr183/Thr172 (ab23875, Abcam), anti-SREBF1 (14088-1-AP, Proteintech), anti-p-ACCα (AF3421, Affinity), and anti-FASN (10624-2-AP, Proteintech) antibodies. Briefly, paraffin sections were heated in citrate buffer for antigen retrieval, 3% hydrogen peroxide solution was used to eliminate endogenous peroxidase, and then sections were blocked with 2% goat serum. After diluting the primary antibody according to the ratio, the sections were placed flat in a wet box and incubated with the primary antibody at 4°C overnight. The next day, incubation was carried out with the secondary antibody of the corresponding species followed by DAB horseradish peroxidase color development according to the manufacturer’s protocols. Image acquisition and analysis can be performed followed by staining the nucleus and sealing the slices. For Hic-5-α-SMA and Hic-5-PTEN fluorescence co-staining, the procedures from antigen retrieval to incubation of secondary antibody were similar with immunohistochemistry. Tyramide diluent (100mL 1× TBST + 100μL 3%H_2_O_2_) was then prepared. Mix 1 mL Tyramide dilution with 2uL CY3-Tyramide to obtain TSA staining working solution. The slices were incubated with TSA staining solution at room temperature for 10 min and washed three times with PBS. The steps of antigen retrieval to TSA staining were subsequently repeated by switching to fluorescent dyes. Next, after staining the nucleus with DAPI and adding fluorescence quencher, the sections were washed and sealed for observation and photographing.

**Metabolomics and RNA sequencing**

The metabolomics and RNA sequencing of livers in Hic-5 KO mice and WT mice fed with HFD or control diet were performed by Majorbio Bio-Pharm Technology Co., Ltd (Shanghai, China). For metabolomic analysis, Principal components analysis (PCA) and Orthogonal partial least squares discriminant analysis (OPLS-DA) were performed at SIMCA (a flexible and comprehensive software for processing metabolomics data). The OPLS-DA model was evaluated using R2 and Q2 values to assess goodness of fit, and the risk of overfitting was examined through 200 permutation tests. Statistical significance (p-value) was determined via univariate analysis (t-test). Metabolites with a variable importance in projection (VIP) > 1, p-value < 0.05, and |fold change| ≥ 2 were considered differentially abundant. For RNA-seq data, principal component analysis (PCA) and differential gene expression analysis were performed in R using the DESeq2 package. P-values were obtained from Wald tests, and the false discovery rate was controlled by applying the Benjamini–Hochberg procedure, resulting in an adjusted p-value. Genes with an absolute log_2_ (fold change) ≥ 1.5 and an adjusted p-value < 0.05 were considered statistically significant.

**Serum levels of transaminase and lipids**

Blood was collected from mice and then centrifuged at 3000rpm for 15 minutes at 4°C. The supernatant is taken and used for the determination of serum transaminase and lipid levels. ALT (C009-2-1, Njjcbio), AST (C010-2-1, Njjcbio), NEFA (A042-2-1, Njjcbio) and TG (A110-1-1, Njjcbio) were measured according to the manufacturer’s protocols.

**Enzyme-linked immune absorbance assay (ELISA)**

Serum inflammatory cytokines TNF-α, IL-6, and IL-1β were detected using ELISA kits (RX202412M, RX203049M, RX203063M, Ruixinbio) according to the manufacturer’s instructions.

**Cell lines and culture**

Human hepatic stellate cell line LX-2 (SCSP-527, NCACC) was cultured in LX-2 Growth Medium (CM-0560, Procell), human HepG2 hepatocyte line (SCSP-510, NCACC) and 293T cell line (SCSP-502, NCACC) were cultured in Dulbecco's modified eagle medium (DMEM) high glucose (SH30022.01, HyClone) which was supplemented with 10% (v/v) fetal bovine serum (10099141C, Gibco), 1% (v/v) penicillin and streptomycin (C0222, Beyotime ).

**Isolation and culture of primary hepatocytes and primary hepatic stellate cells**

Primary hepatocytes and hepatic stellate cells were obtained by in situ reverse perfusion Mice were anesthetized and opened the abdomen. Move the intestine to the left side of the mouse as far as possible to allow adequate visualization of the inferior vena cava (IVC) and portal vein. Then, a catheter was inserted into the IVC. The assistant pulls the sternum proximally, and the operator closes the second porta hepatis with a vascular clamp. Immediately, liver was perfused with EGTA solution and portal vein was cut. When the liver appeared pale, it was switched to Pronase E (P8360, Solarbio) solution for perfusion until the liver appeared to be snowy. Subsequently, collagenase IV (C8160，Solarbio) solution continued to be used for perfusion. After perfusion was completed, the liver was placed in a petri dish and disaggregated with scissors. The tissue dissociation solution was transferred into a 50 ml centrifuge tube and digested by shaking at 37°C for 20 min. The cells were filtered through a 70μm filter and centrifuged at 50 g/4℃ for 1min, and the precipitate was hepatocytes. Hepatocytes were inoculated in flasks which were pre-plated with rat tail tendon collagen type I (C8065, Solarbio) and cultured in DMEM (10% FBS, 1% penicillin and streptomycin), while the supernatant was continued for gradient centrifugation to obtain hepatic stellate cells. Briefly, cells in the supernatant were gained after centrifugation and resuspended with PBS. Then add 50% percoll, 25% percoll and cell suspension slowly and sequentially into a 15 ml tube, followed by centrifugation at 1380 g/4°C for 20 min. Hepatic stellate cells were located at the junction of percoll where looks like a white ring. The cells were extracted from the white ring and then re-suspended and cultured in specialized medium (PriMed-iCell-009, iCell).

**Palmitic acid and oleic acid exposure**

To simulate the high-fat environment *in vitro*, the LX-2 and HepG2 cell lines, the primary hepatocytes and hepatic stellate cells were exposed with 0.5 mM palmitic acid and 1 mM oleic acid (Kunchuang biotechnology, KT004). BSA was used as control.

**Cell transfection**

The Hic-5 adenovirus vector and Hic-5 lentiviral vector, which were constructed by Hanbio (Shanghai, China) were used to overexpress Hic-5 in primary hepatic stellate cells and LX-2 cells respectively. Adenovirus targeting Hic-5 (Ad-Hic-5) and its negative control (Ad-Ctrl) infected primary hepatic stellate cells at a multiplicity of infection (MOI) of 40 and in the presence of 4 μg/ml polybrene for 24 hours. Lentiviral targeting Hic-5 (LV-Hic-5) and its negative control (LV-Ctrl) infected LX-2 cells at a MOI of 15 and in the presence of 8 μg/ml polybrene for 24 hours. Furthermore, LX-2 cells were transfected with siRNA to knockdown the expression of Hic-5 and SP1, HepG2 cells were transfected with siRNA to knockdown the expression of EP1, EP2, EP3 and EP4. Each of siRNA was constructed by RiboBio (Guangzhou, China), and 200 pmol of each siRNA infected cells in the presence of 250 μl Lipofectamine 8000 (C0533, Beyotime). Total proteins were prepared and the efficiency of overexpression or knockdown was checked by Western blot.

**Cell co-culture**

We constructed co-culture of LX-2 and HepG2 as well as primary hepatic stellate cells and hepatocytes. LX-2 cells were seeded on the co-culture chamber (3450, Corning) and HepG2 cells were below LX-2. The co-cultured system was supplied with palmitic acid and oleic acid medium 12 hours after seeded and continued incubation for 24 hours. For co-culture of primary hepatic stellate cells and hepatocytes, we used a conditioned media co-culture method. The culture media was collected 24 hours after incubation of primary stellate cells in palmitic acid and oleic acid medium, and the primary hepatocytes were directly cultured in supernatant after centrifugation to remove the precipitation.

**PGE2 and 2B5 treatment**

Human recombinant PGE2 (HY-101952, MCE) was administered in primary mice hepatocytes and HepG2 cells at a concentration of 20 ng/ml. Sterile water was used as vehicle. As for the co-culture system of Hic-5-overexpressing pHSCs and pHep, 10 μg/mL neutralizing anti-PGE antibody (2B5, Cayman Chemical) was administered.

**PGE2 measurement in serum and culture supernatant**

PGE2 levels were determined in serum and the culture medium of LX-2 cells and primary hepatic stellate cells by PGE2 Assay Kit (RX105367H, Ruixinbio). The culture medium was collected and spin at 4000rpm g for 20 min and assayed directly. Then, 50 µl of sample or standard to were added to wells of a 96-well plate, followed by 50 µl of biotinylated antigen to each well. After the incubation for 60 minutes at 37℃, we washed plate for three times. Next, 50 µl of HRP-conjugate was added to each well and continue incubation for 30min at 37℃. The plate was washed for three times and 50ul of colorant A and B were added to each well respectively. Finally, the absorbance was measured at 450nm. PGE2 levels were calculated against the standard curve concentration.

**PGE2 receptor inhibitor intervention**

The inhibitor of four PGE2 receptors SC-51322 (HY-108562, MCE), PF-04418948 (HY-18966, MCE), L-798106 (HY-15274, MCE) and L-161982 (HY-108559, MCE) were used to identify the involved receptor. The concentrations are 5 μM, 2 μM, 200 nM, and 10 μM, respectively. As for the animal experiments with L-161982, intraperitoneal injection was administered at a concentration of 5 mg/kg.

**Real-time quantitative PCR (RT-qPCR)**

Liver tissue or cell total RNA was extracted using the TRIpure FastPure Cell/Tissue Total RNA Isolation Kit (RC101-01, Vazyme), and cDNA was synthesized using HiScript® III all-in-one real-time (RT) SuperMix Perfect for quantitative polymerase chain reaction (R333-01, Vazyme). Real-time fluorescence quantitative polymerase chain reaction (RT-qPCR) was performed using ChamQ Universal SYBR qPCR Master Mix (Q711-02, Vazyme). The expression levels of target genes were normalized to those of GAPDH.

**Table S1. Primers used for gene amplification.**

| **Name** | **Sequence 5´→ 3´** | **Supplier** |
| --- | --- | --- |
| Human-Hic-5 (Forward Primer) | TACAGCACGCTATGCAAGCC | Sangon Biotech |
| Human-Hic-5 (Reverse Primer) | GCAACCGATCTAGCTCACAGAG | Sangon Biotech |
| Human-EP1 (Forward Primer) | AGCTTCTCGCTATCATGGTGG | Sangon Biotech |
| Human-EP1 (Reverse Primer) | AAGAGGCCAAGCACTTGGC | Sangon Biotech |
| Human-EP2 (Forward Primer) | GAAACCTCTTCCCCAAAGCAAA | Sangon Biotech |
| Human-EP2 (Reverse Primer) | GACTGAACGCATTAGTCTCAGAA | Sangon Biotech |
| Human-EP3 (Forward Primer) | CGCCTCAACCACTCCTACAC | Sangon Biotech |
| Human-EP3 (Reverse Primer) | CACACCCATCCCCAATCCTC | Sangon Biotech |
| Human-EP4 (Forward Primer) | CCCCCGCTCATCTTCATCTT | Sangon Biotech |
| Human-EP4 (Reverse Primer) | CCCACATACCACCCTCTACAA | Sangon Biotech |
| Mouse-Hic-5 (Forward Primer) | ATGTCACGGTTAGGGGCTC | Sangon Biotech |
| Mouse-Hic-5 (Reverse Primer) | GGCTTGCATACTGTGCTGTATAG | Sangon Biotech |
| Mouse-ACCα (Forward Primer) | CTTCCTGACAAACGAGTCTGG | Sangon Biotech |
| Mouse-ACCα (Reverse Primer) | CTGCCGAAACATCTCTGGGA | Sangon Biotech |
| Mouse-FASN (Forward Primer) | GGAGGTGGTGATAGCCGGTAT | Sangon Biotech |
| Mouse-FASN (Reverse Primer) | TGGGTAATCCATAGAGCCCAG | Sangon Biotech |

**Western blot (WB)**

Total proteins were prepared as our previously described. Proteins were extracted from tissues or cells using RIPA lysis buffer (P0013B, Beyotime). Then, equal amounts of protein were separated by 10% SDS-PAGE and transferred to PVDF membrane later. The PVDF membranes were blocked with TBST containing 5% nonfat milk for 1 h, and washed using TBST three times. Incubation with primary antibodies were performed overnight at 4℃ with anti-Hic-5 (10565-1-AP, Proteintech, 1:1000), anti-AMPK (10929-2-AP, Proteintech, 1:5000), anti-p-AMPK Thr183/Thr172 (ab23875, Abcam, 1:500), anti-SREBP1 (14088-1-AP, Proteintech, 1:1000), anti-FASN (10624-2-AP, Proteintech, 1:5000), anti-PTGES (ab180589, Abcam, 1:1000), anti-PTGS2 (66351-1-Ig, Proteintech, 1:1000), anti-SP1 (21962-1-AP, Proteintech, 1:2000), anti-p-SP1 Thr739 (PA5-104771, Invitrogen, 1:500), anti-PTEN (ab267787, Abcam, 1:1000), anti-p-PTEN Ser380/Thr382/383 (9549T, CST, 1:1000), anti-c-Src (40790-1, SAB, 1:500), anti-p-c-Src Tyr416 (2101S, CST, 1:1000), anti-Flag Tag (20543-1-AP, Proteintech, 1:20000), anti-HA Tag (66006-2-Ig, Proteintech, 1:10000), anti-GAPDH (60004-1-Ig, Proteintech, 1:5000), anti-β-actin (66009-1-Ig, Proteintech, 1:5000). Afterward, the membranes were incubated with secondary antibodies for 1 h at room temperature. Finally, they were detected by the enhanced chemiluminescence (ECL) detection system.

**Co-Immunoprecipitation (Co-IP)**

Co-IP of Hic-5 and binding proteins was performed using LX-2 and 293T cells. As our previously described, cells were collected and lysed in Lysis Buffer for IP (P0013, Beyotime). Then, 40 μl of Protein A+G Agarose (P2012, Beyotime) were added to each 1 ml of total protein and shaking slowly for 1 hour at 4°C. Subsequently, the tubes were centrifuged at 2500 rpm for 5 minutes and the supernatant was used for following immunoprecipitation. Next, add 1 µg of primary antibody for immunoprecipitation to the supernatant and shake slowly at 4°C overnight. On the second day, 40 μl of fully suspended Protein A+G Agarose was added, and the precipitation was obtained by centrifugation after slow shaking at 4℃ for 2 hours. The precipitation was used for Western blot.

**Supplementary figures**

**Figure S1**

**
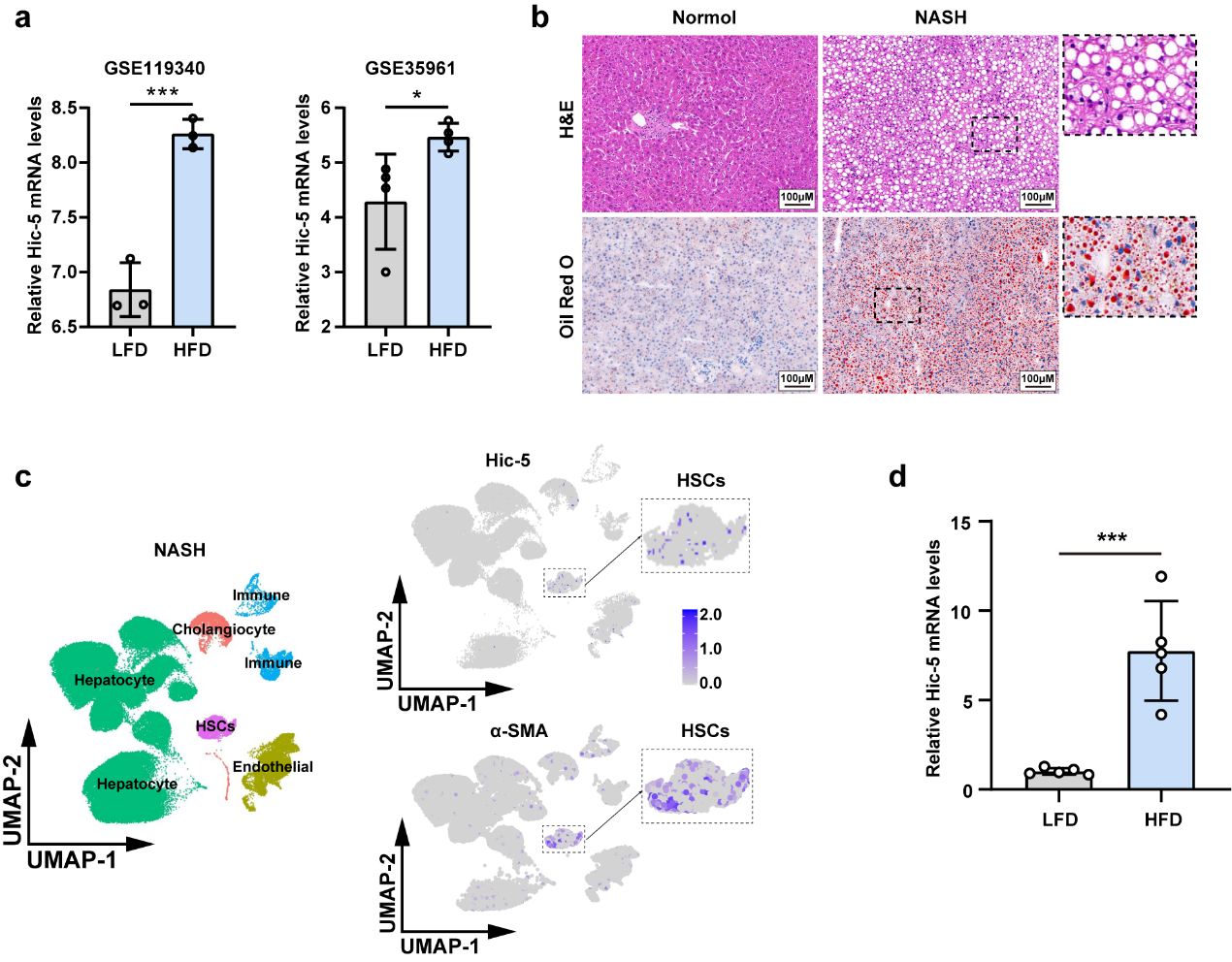
**

**Figure S1. Hic-5 is upregulated in mouse models of NASH and is highly expressed in hepatic stellate cells in the liver of patients with NASH.** (**a**) The mRNA expression levels of Hic-5 in mice transcriptome datasets (GSE119340 and GSE35961). (**b**) Representative H&E and Oil red O staining images in the liver tissues from patients with NASH and normal controls. (**c**) Single-cell RNA sequencing reveals the expression of Hic-5 and co-localized with α-SMA in liver cells of patients with NASH. (**d**) The mRNA expression levels of Hic-5 in mouse models of NASH (n=5/group). Data are expressed as mean ± SD. **p <0.05, **p <0.01, ***p <0.001*.

**Figure S2**

**
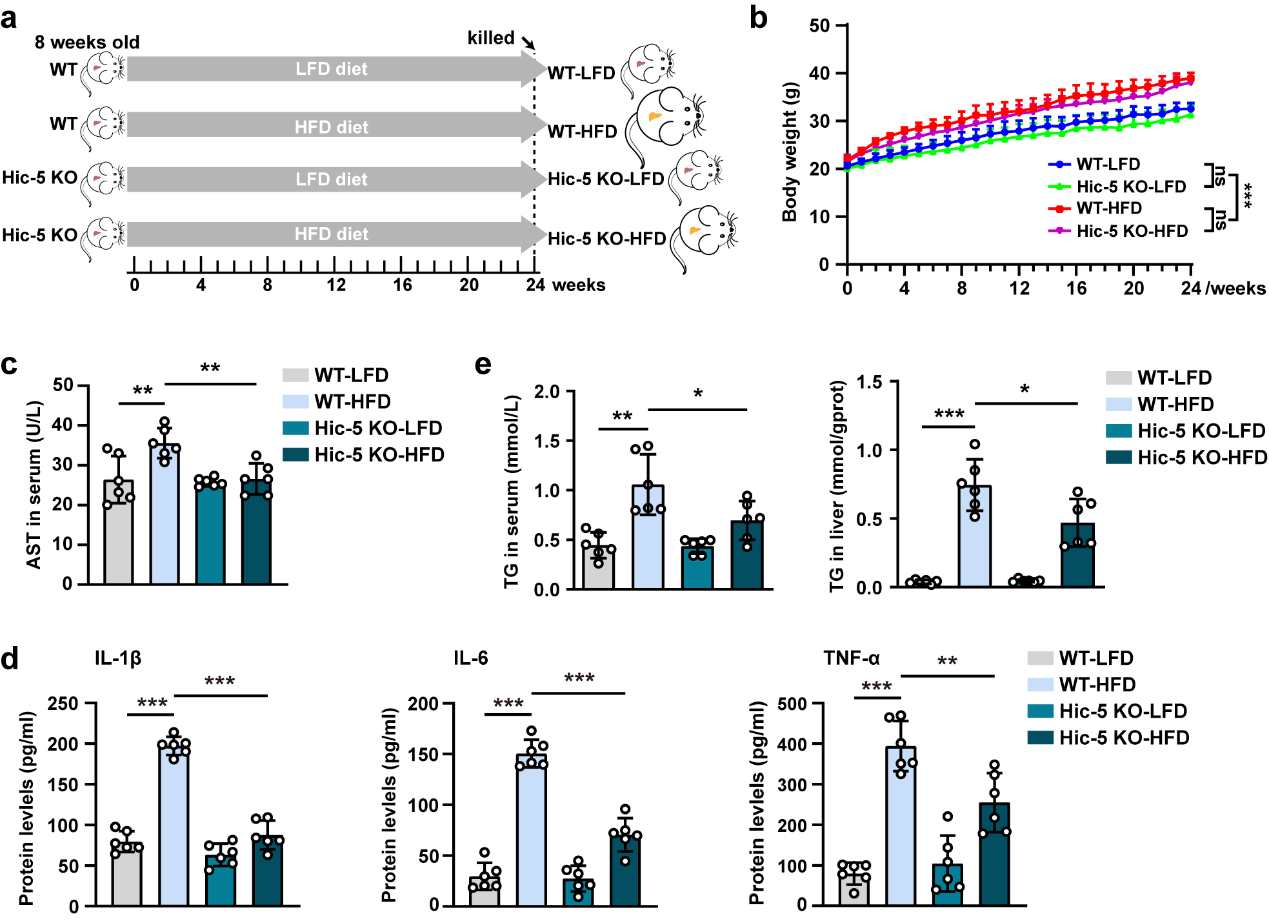
**

**Figure S2. Hic-5 deficiency alleviates NASH phenotype.** (**a**) The schematic diagram of establishing NASH models by feeding Hic-5 KO and WT mice with HFD or normal diet. (**b**) The body weight of mice (n=6/group). (**c, d**) Serum AST, IL-1β, IL-6 and TNF-α levels (n=6/group). (**e**) Serum and liver tissue TG levels (n=6/group). Data are expressed as mean ± SD. **p <0.05, **p <0.01, ***p <0.001*.

**Figure S3**

**
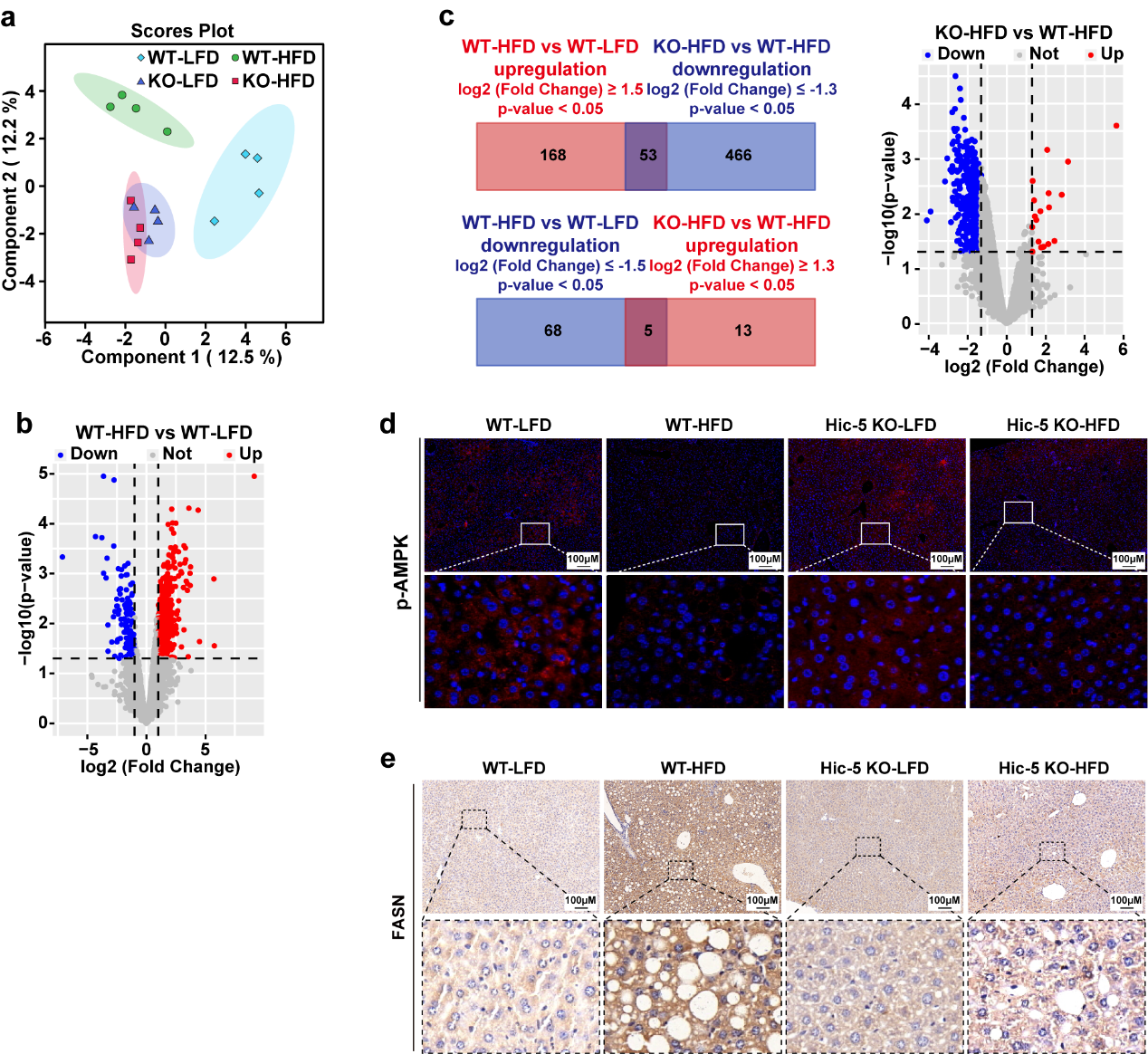
**

**Figure S3. Hic-5 deficiency upregulates hepatic phosphorylated AMPK signaling pathway.** (**a**) PCA analysis of gene expression in the four sample groups. (**b**) Volcano maps of differentially expressed genes. (**c**) The Venn diagram showed hub genes identified from differentially expressed genes. (**d**) The fluorescence staining of p-AMPK in Hic-5 KO and WT mice liver tissues fed with HFD or LFD diet. (**e**) Representative IHC of FASN from liver tissues.

**Figure S4**

**
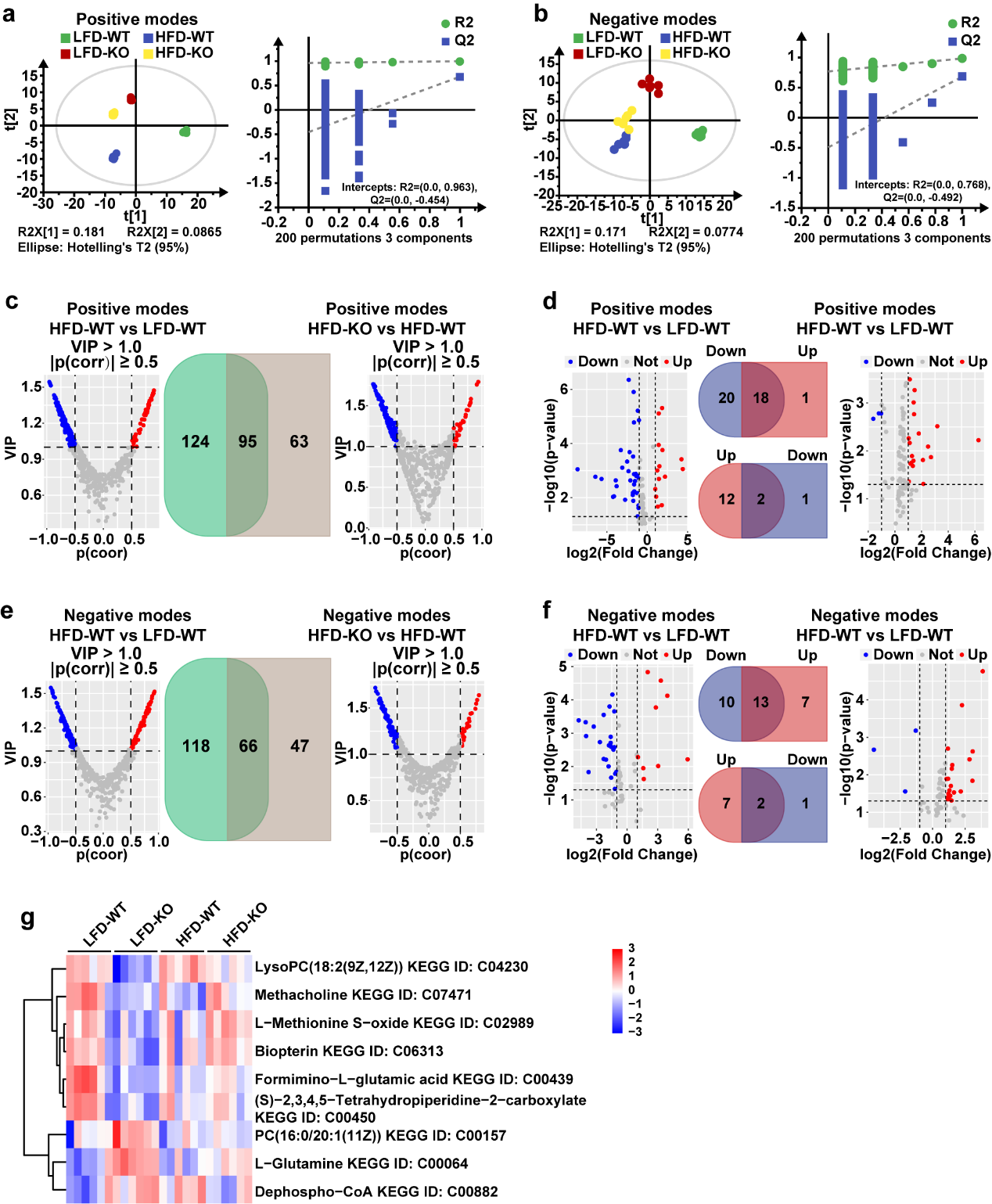
**

**Figure S4. Hic-5 deficiency improves hepatic fatty acid metabolism in mouse models of NASH.** (**a**) Positive‐mode OPLS‐DA score plot. (**b**) The corresponding validation plot based on 200 times permutation tests demonstrated the robustness of the OPLS-DA model in a positive mode. (**c**) Negative‐mode OPLS‐DA score plot. (**d**) The corresponding validation plot based on 200 times permutation tests demonstrated the robustness of the OPLS-DA model in a negative mode. (**e**) Volcano plot of differentially expressed metabolites (DEMs) with VIP>1 and |P(corr)| ≥ 0.5 in positive modes. (**f**) Volcano plot of hub DEMs with |log2(Fold Change)| > 1.0 and p-value< 0.05 in positive modes. (**g**) Volcano plot of differentially expressed metabolites (DEMs) with VIP>1 and |P(corr)| ≥ 0.5 in negative modes. (H) Volcano plot of hub DEMs with |log2(Fold Change)| > 1.0 and p-value< 0.05 in negative modes. (I) Heat map of hub DEMs.

**Figure S5**


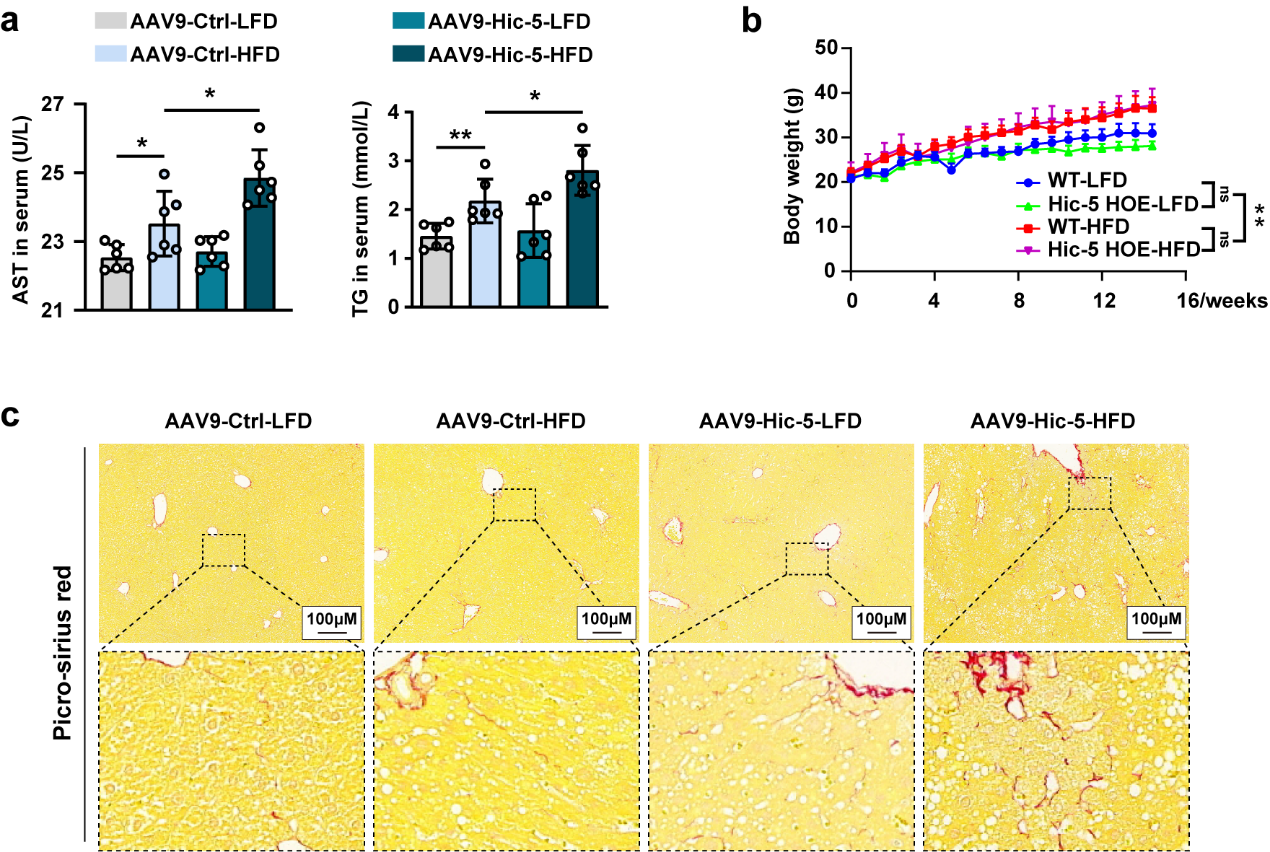


**Figure S5. Hic-5 upregulation in hepatic stellate cells aggravates NASH phenotype.** (**a**) Serum AST and TG levels (n=6/group). (**b**) The body weight of Hic-5 HOE mice fed with HFD or LFD diet (n=6/group). (**c**) Representative Sirius red staining of liver tissues. Data are expressed as mean ± SD. **p <0.05, **p <0.01, ***p <0.001*.

**Figure S6**


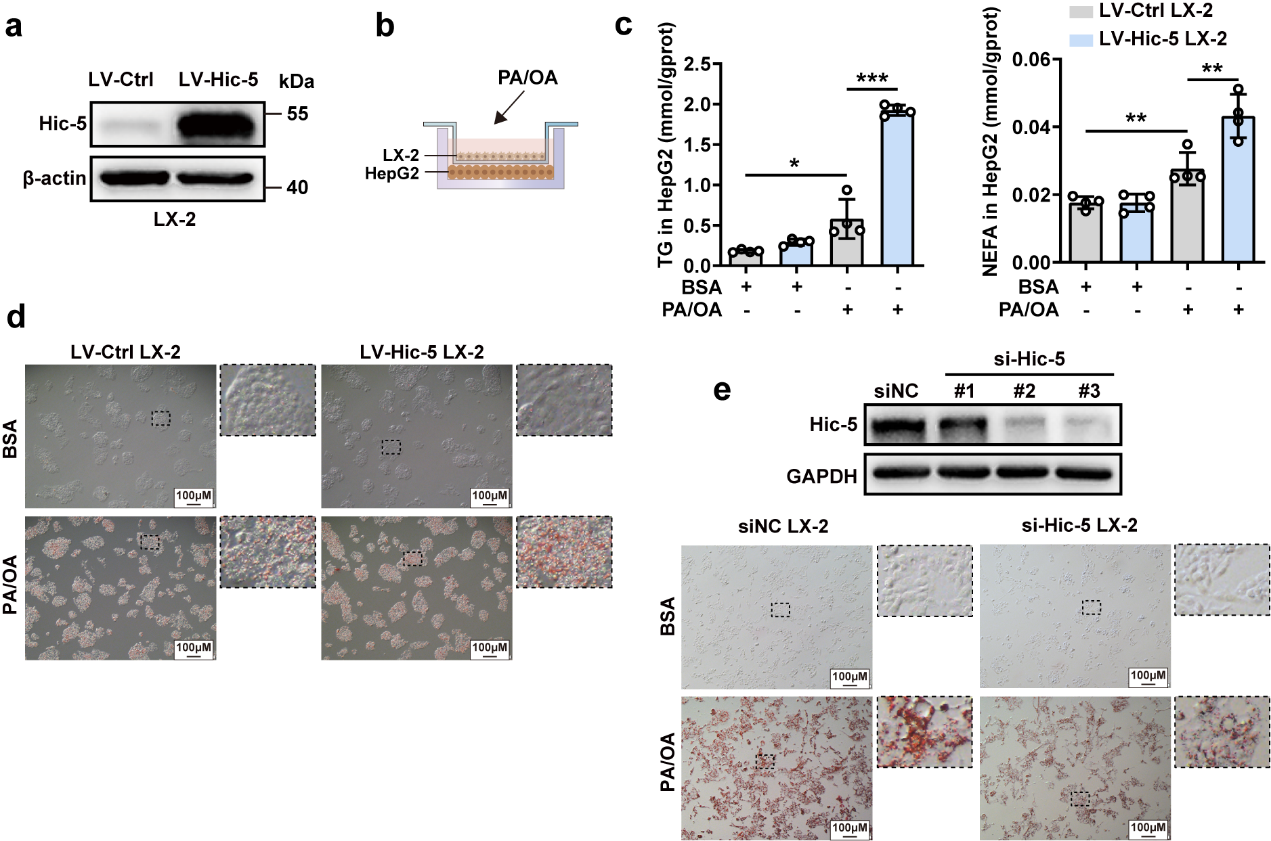


**Figure S6. Hic-5 in hepatic stellate cells promotes hepatocellular fatty acid synthesis.** (**a**) Successful overexpression of Hic-5 in LX-2 using LV-Hic-5 was confirmed by WB. (**b**) Schematic diagram of co-culture system of LX-2 and HepG2. (**c, d**) The NEFA, TG levels in HepG2 and representative Oil red O staining of HepG2 after co-cultured with LV-Hic-5 LX-2 (n=4/group). (**e**) Knockdown of Hic-5 using siRNA was detected by WB, and representative Oil red O staining of HepG2 after co-cultured with si-Hic-5 LX-2. Data are expressed as mean ± SD. **p <0.05, **p <0.01, ***p <0.001*.

**Figure S7**


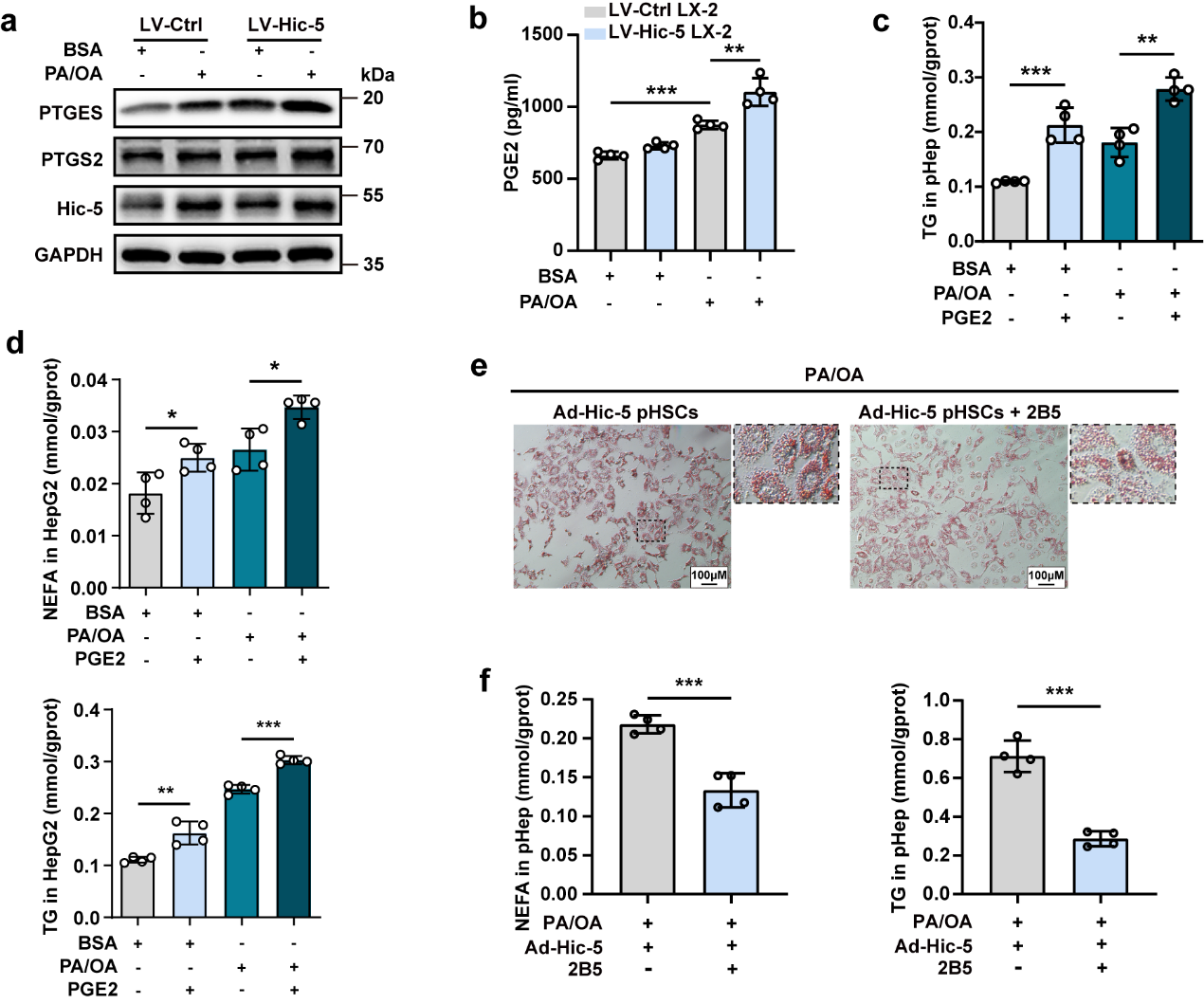


**Figure S7. Hic-5 promotes PEG2 expression and secretion.** (**a**) The expression of PTGES and PTGS2 in LV-Hic-5 LX-2 exposed to PA/OA or not. (**b**) The PGE2 levels from supernatant of LX-2 (n=4/group). (**c**) The TG levels in pHep treated with PGE2 (n=4/group). (**d**) The NEFA and TG levels in HepG2 treated with PGE2 (n=4/group). (**e, f**) Representative Oil red O staining of the co-culture system with overexpressed Hic-5 intervened by PGE2 neutralizing antibody 2B5, and NEFA, TG levels (n=4/group) in pHep. Data are expressed as mean ± SD. **p <0.05, **p <0.01, ***p <0.001*.**Figure S8**


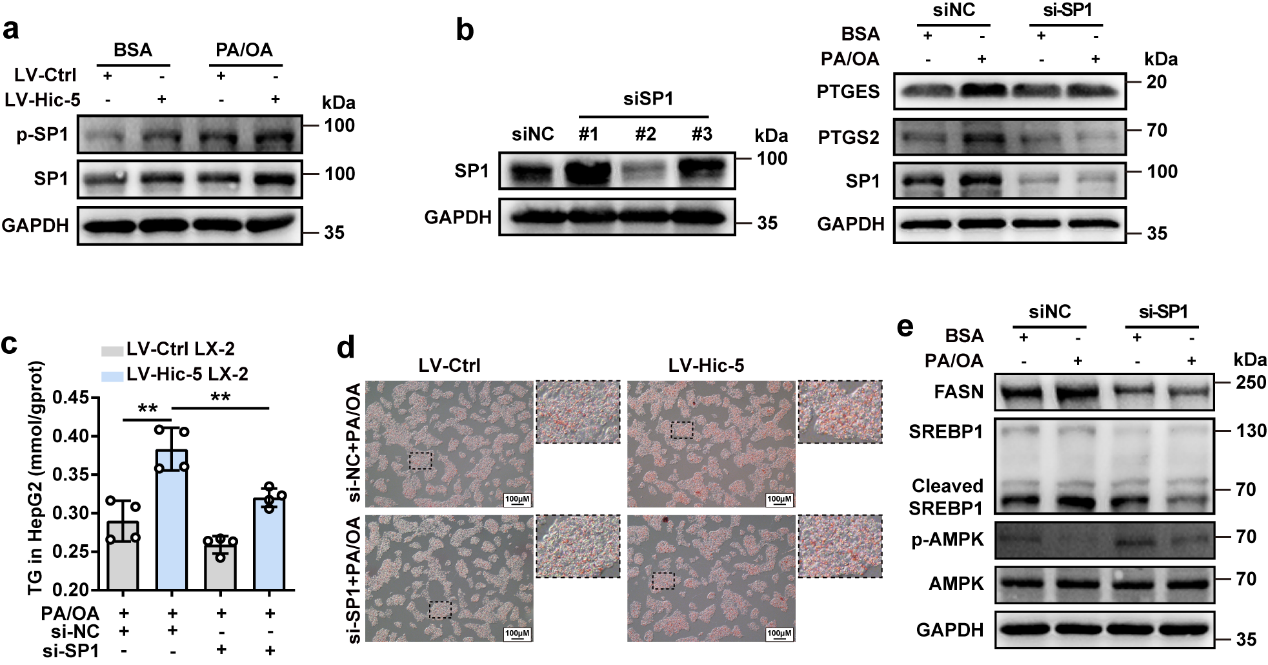


**Figure S8. Hic-5 promotes PEG2 expression and secretion through SP1 in hepatic stellate cells.** (**a**) The expression of SP1 and p-SP1 in LV-Hic-5 LX-2 exposed to PA/OA or not. (**b**) The expression of PTGES and PTGS2 in LV-Hic-5 LX-2 after knockdown of SP1. (**c-e**) The TG levels (n=4/group), representative Oil red O staining and the expression of p-AMPK, SREBP1 and FASN in HepG2 were detected after knockdown of SP1 in LV-Hic-5 LX-2 cells and co-cultured with HepG2. Data are expressed as mean ± SD. **p <0.05, **p <0.01, ***p <0.001*.

**Figure S9**


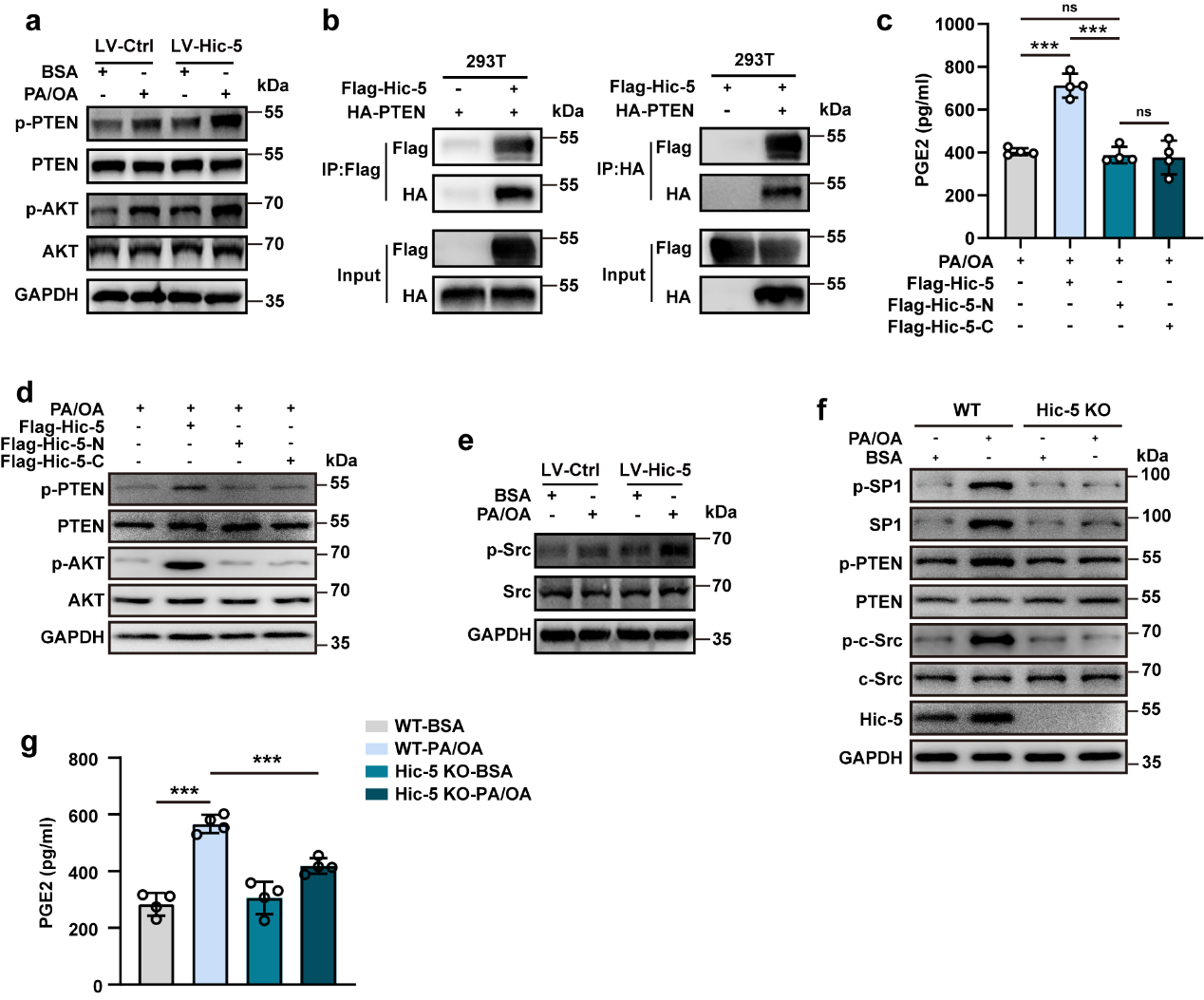


**Figure S9.** **Hic-5 binds to PTEN and c-Src.** (**a**) Phosphorylation of AKT and PTEN in Hic-5 overexpressed LX-2 cells were detected by WB. (**b**) Co-IP results of Hic-5 and PTEN in 293T cells. (**c, d**) The PGE2 levels (n=4/group) and phosphorylation of PTEN and AKT in LX-2 cells were detected when transfected N-terminal or C-terminal of Hic-5. (**e**) Phosphorylation of c-Src in Hic-5 overexpressed LX-2 cells were detected by WB. (**f, g**) The expression of c-Src-PTEN-SP1 axis and the PGE2 levels (n=4/group) in pHSCs from Hic-5 KO mice when exposed to PA/OA or not were detected. Data are expressed as mean ± SD. **p <0.05, **p <0.01, ***p <0.001*.**Figure S10**


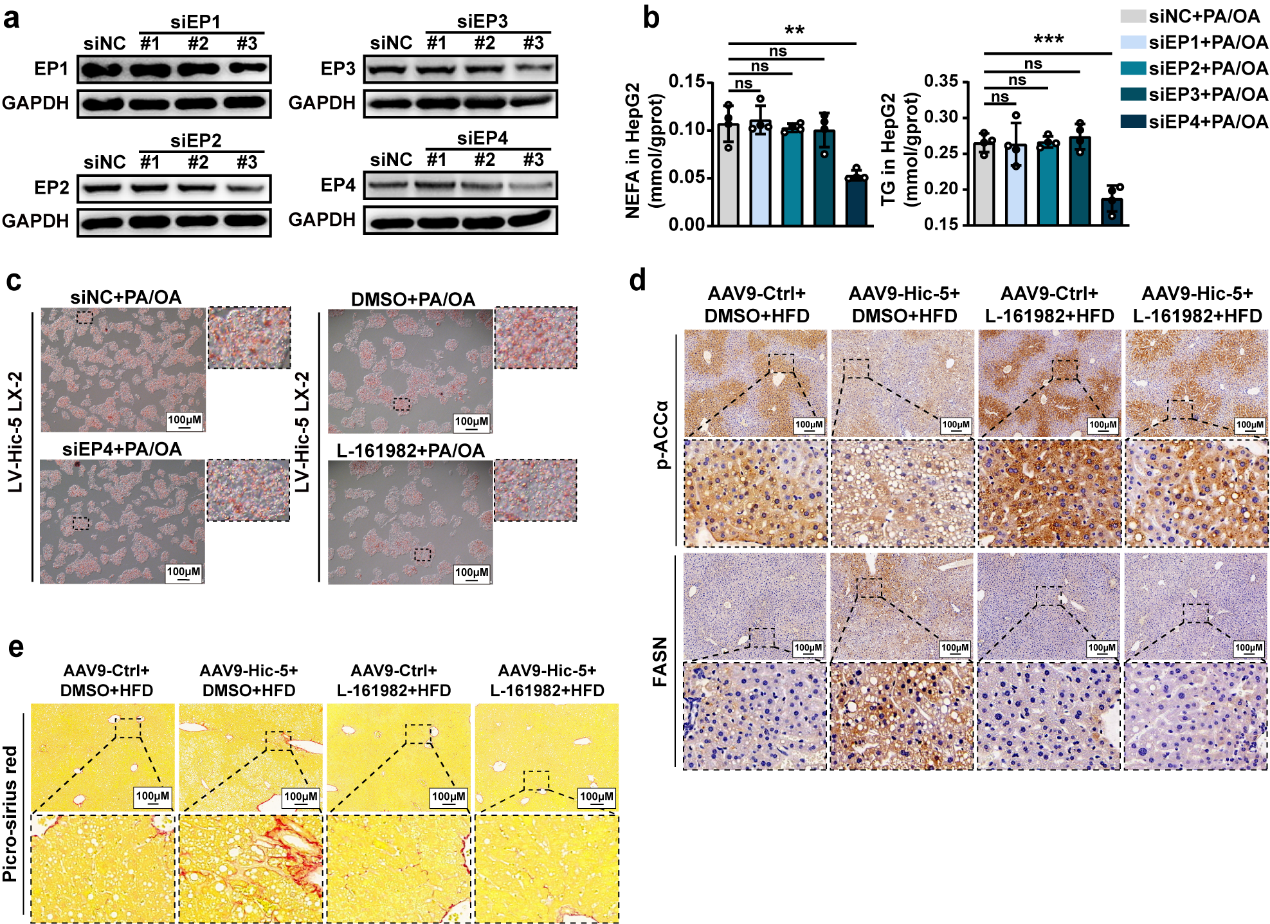


**Figure S10. Hic-5 promotes hepatocellular fatty acid synthesis through the PGE2-EP4 axis.** (**a**) Knockdown of EP1, EP2, EP3 and EP4 using siRNA was detected by WB. (**b**) The NEFA and TG levels in HepG2 after knockdown of EP1, EP2, EP3 and EP4, and co-cultured with LV-Hic-5 LX-2 cells (n=4/group). (**c**) Oil red O staining of HepG2 when treated with siRNA and L-161982, and co-cultured with LV-Hic-5 LX-2 cells. (**d, e**) Representative IHC of P-ACCα, FASN and Sirius red staining of liver tissues from HFD-fed Hic-5 HOE mice and treated with EP4 inhibitor L-161982. Data are expressed as mean ± SD. **p <0.05, **p <0.01, ***p <0.001*.

**Table S1. Primers used for gene amplification.**

| **Name** | **Sequence 5´→ 3´** | **Supplier** |
| --- | --- | --- |
| Human-Hic-5 (Forward Primer) | TACAGCACGCTATGCAAGCC | Sangon Biotech |
| Human-Hic-5 (Reverse Primer) | GCAACCGATCTAGCTCACAGAG | Sangon Biotech |
| Human-EP1 (Forward Primer) | AGCTTCTCGCTATCATGGTGG | Sangon Biotech |
| Human-EP1 (Reverse Primer) | AAGAGGCCAAGCACTTGGC | Sangon Biotech |
| Human-EP2 (Forward Primer) | GAAACCTCTTCCCCAAAGCAAA | Sangon Biotech |
| Human-EP2 (Reverse Primer) | GACTGAACGCATTAGTCTCAGAA | Sangon Biotech |
| Human-EP3 (Forward Primer) | CGCCTCAACCACTCCTACAC | Sangon Biotech |
| Human-EP3 (Reverse Primer) | CACACCCATCCCCAATCCTC | Sangon Biotech |
| Human-EP4 (Forward Primer) | CCCCCGCTCATCTTCATCTT | Sangon Biotech |
| Human-EP4 (Reverse Primer) | CCCACATACCACCCTCTACAA | Sangon Biotech |
| Mouse-Hic-5 (Forward Primer) | ATGTCACGGTTAGGGGCTC | Sangon Biotech |
| Mouse-Hic-5 (Reverse Primer) | GGCTTGCATACTGTGCTGTATAG | Sangon Biotech |
| Mouse-ACCα (Forward Primer) | CTTCCTGACAAACGAGTCTGG | Sangon Biotech |
| Mouse-ACCα (Reverse Primer) | CTGCCGAAACATCTCTGGGA | Sangon Biotech |
| Mouse-FASN (Forward Primer) | GGAGGTGGTGATAGCCGGTAT | Sangon Biotech |
| Mouse-FASN (Reverse Primer) | TGGGTAATCCATAGAGCCCAG | Sangon Biotech |
